# Novel Hybrid Compounds Containing Benzofuroxan and Aminothiazole Scaffolds: Synthesis and Evaluation of Their Anticancer Activity

**DOI:** 10.3390/ijms22147497

**Published:** 2021-07-13

**Authors:** Elena Chugunova, Gabriele Micheletti, Dario Telese, Carla Boga, Daut Islamov, Konstantin Usachev, Alexander Burilov, Alena Tulesinova, Alexandra Voloshina, Anna Lyubina, Syumbelya Amerhanova, Tatiana Gerasimova, Aisylu Gilfanova, Victor Syakaev

**Affiliations:** 1 FRC Kazan Scientific Center, Arbuzov Institute of Organic and Physical Chemistry, Russian Academy of Sciences, 420088 Kazan, Russia; daut1989@mail.ru (D.I.); burilov_2004@mail.ru (A.B.); microbi@iopc.ru (A.V.); aplyubina@gmail.com (A.L.); syumbelya07@mail.ru (S.A.); tatyanagr@gmail.com (T.G.); aisylurafikovna@mail.ru (A.G.); vsyakaev@iopc.ru (V.S.); 2 Laboratory of Plant Infectious Diseases, FRC Kazan Scientific Center of Russian Academy of Sciences, 420111 Kazan, Russia; 3 Department of Industrial Chemistry ‘Toso Montanari’, Alma Mater Studiorum—Universita di Bologna Viale Del Risorgimento, 40136 Bologna, Italy; dario.telese2@unibo.it; 4 Kazan Institute of Biochemistry and Biophysics, FRC Kazan Scientific Center of Russian Academy of Sciences, 420111 Kazan, Russia; k.usachev@mail.ru; 5 Institute of Chemical Engineering and Technology, Kazan National Research Technological University, 420015 Kazan, Russia; tulessinova.a@yandex.ru

**Keywords:** benzofuroxan, aminothiazole, anticancer activity, apoptosis, quantum chemicalcal culations

## Abstract

A series of novel hybrid compounds containing benzofuroxan and 2-aminothiazole moieties are synthesized via aromatic nucleophilic substitution reaction. Possible reaction pathways have been considered quantum-chemically, which allowed us to suggest the most probable products. The quantum chemical results have been proved by X-ray data on one compound belonging to the synthesized series. It was shown that the introduction of substituents to both the thiazole and amine moieties of the compounds under study strongly influences their UV/Vis spectra. Initial substances and obtained hybrid compounds have been tested in vitro as anticancer agents. Target compounds showed selectivity towards M-HeLa tumor cell lines and were found to be more active than starting benzofuroxan and aminothiazoles. Furthermore, they are considerably less toxic to normal liver cells compared to Tamoxifen. The mechanism of action of the studied compounds can be associated with the induction of apoptosis, which proceeds along the mitochondrial pathway. Thus, new hybrids of benzofuroxan are promising candidates for further development as anticancer agents.

## 1. Introduction

Currently, oncological diseases are one of the most serious problems facing humanity, requiring the development of new drugs and methods of treatment. Cases of improving methods of therapy for oncological diseases, leading to a dramatic improvement in treatment results, are extremely rare, and most of the drugs used to treat cancer are toxic to the body as a whole [1].

There are several different classes of anticancer drugs based on their mechanisms of action: alkylating agents, antimetabolites, mitotic inhibitors, antineoplastic antibiotics, hormonal agents, and miscellaneous [2,3]. All anticancer drugs are not strictly specific; they act not only on tumor cells but also on normal cells, primarily on tissues with rapid proliferation (bone marrow, gastrointestinal mucosa) [4]. This determines the presence of toxic effects, which are often very serious, in almost all anticancer drugs.

The end result of the action of all antitumor drugs is the inhibition of cell proliferation and the death of tumor cells (tumor regression) [5]. This effect is achieved by acting on various targets in the cell: DNA; RNA; enzymes necessary for normal DNA replication and repair; pathways for the intracellular transduction of mitogenic signals; the mitotic apparatus of the cell. As a result of these influences, apoptosis develops—programmed cell death. At the same time, the specific mechanisms for achieving these effects for different drugs within the same group may differ significantly, which is determined by the peculiarities of the chemical structure and metabolism of these compounds. For a number of drugs of different groups, the effect on several targets is characteristic.

Among anticancer drugs of the new generation, promising drugs are those that can selectively induce the death of cancer cells with minimal toxic effects in relation to healthy cells of the body [6]. Such selectivity can be based on the selective induction of death processes in cancer cells: apoptosis, autophagy, or necroptosis [7]. The search for such compounds is the key goal of our research.

2-Aminothiazoles, cyclic thiourea derivatives, are of great importance for pharmaceutical production, biochemistry, engineering, clinical and experimental medicine. 2-Aminothiazole derivatives are used as disperse dyes in cotton industries [8], and mercaptothiazoles are vulcanization accelerators in the rubber industry [9]. These compounds showed high anti-inflammatory, analgesic, antioxidant [10], antiviral [11], antimycobacterial [12], antiplasmodial [13], anticancer [14], neuroprotective and anti-inflammatory properties [15] and other types of activity.

**Figure 1:**
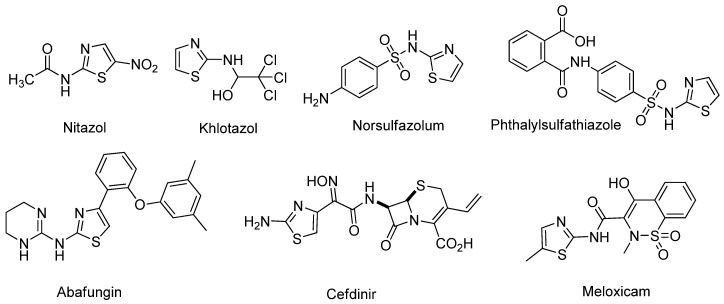
Derivatives of 2-aminothiazole used in medicine and veterinary medicine.

Derivatives of 2-aminothiazole, such as Norsulfazolum, Phthalylsulfathiazole, Khlotazol, Nitazol, Abafungin, Cefdinir, Meloxicam are widely used in medicine and veterinary medicine as a nonsteroidal anti-inflammatory and antimicrobial drugs (Figure 1).

**Figure 2:**
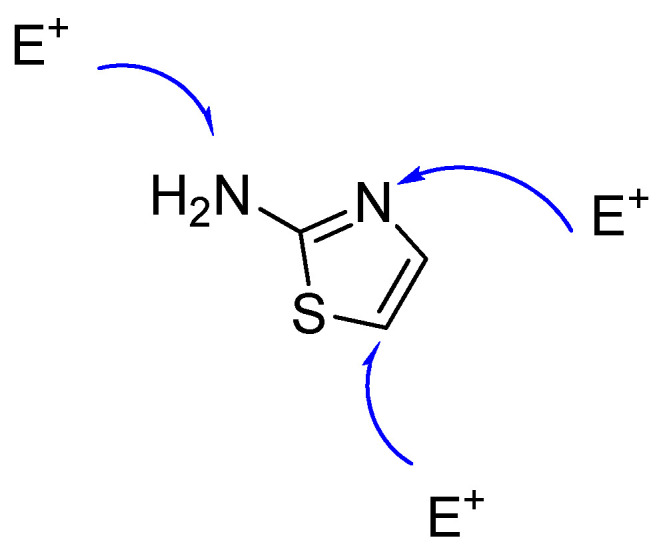
Three possible ways of the reaction between 2-aminothiazole and electrophiles.

In addition, the 2-aminothiazole molecule has a large number of reaction centers (endocyclic nitrogen atom, exocyclic NH_2_ and the carbon atom in Position 5, Figure 2), which prompts additional interest of research chemists in this compound.

Our research group has been conducting research on the reactions of superelectrophilic benzofuroxans with various nucleophiles for a long time. Research was designed integrally, with direction dedicated to the synthesis of hybrid compounds [16,17,18]. It has been shown that the introduction of benzofuroxan fragments into the molecules of various pharmacophores leads to an increase in the biological activity or a decrease in the toxicity of the obtained products. Earlier, we made some studies on the interaction of benzofuroxans with aminothiazole derivatives [19,20,21]. Moreover, the study of such reactions is of interest to other research groups [22,23]. In the present study, we not only carried out a comprehensive study of the reactions of superelectrophilic 7-chloro-4,6-dinitrobenzofuroxan with 2-aminothiazole derivatives using NMR spectroscopy, X-ray diffraction analysis, UV-spectroscopy and quantum chemical calculations, but also studied the anticancer activity of the compounds obtained.

## 2. Results and Discussion

### 2.1. Chemistry

#### 2.1.1. Quantum Chemical Calculations

**Scheme 1:**
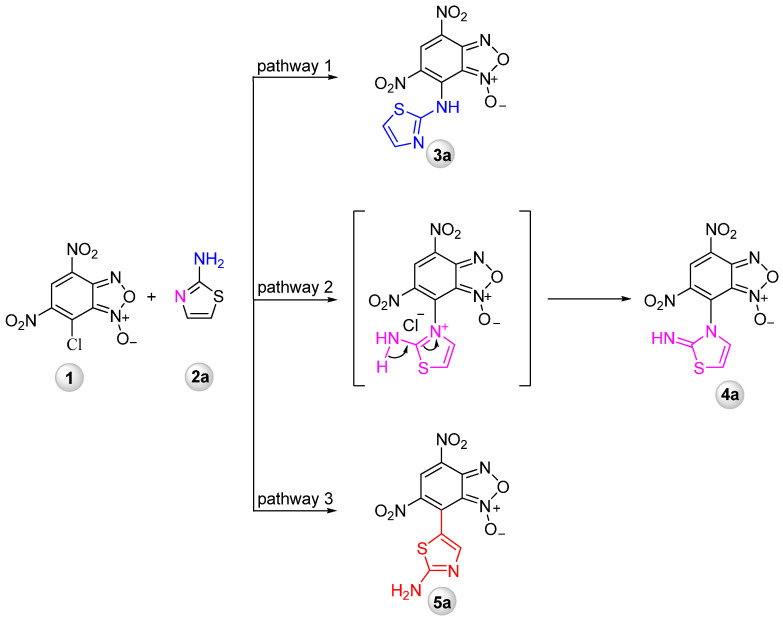
Three possible ways for reaction of 7-chloro-4,6-dinitrobenzofuroxan **1** with 2-aminothiazole derivatives.

To shed light on the peculiarities of the interaction of 7-chloro-4,6-dinitrobenzofuroxan **1** with aminothiazoles **2**, the quantum-chemical study of these reactions has been carried out. On the example of the simplest representative of benzofuroxan and aminothiazole hybrids with R=R_2_=R_3_=H **3a**, three possible ways have been considered for aromatic nucleophilic substitution reaction: Pathway 1—attack of electrophile on the exocyclic NH_2_, Pathway 2—attack of an electrophile on the endocyclic nitrogen atom and Pathway 3—attack on the carbon atom in Position 5 (Scheme 1).

It is well known that 2-aminothiazole reacts with 2,4-dinitrofluorobenzene first with the endocyclic nitrogen atom [24]. Analysis of transition states for three considered ways shows the highest barrier for Pathway 3 (18.3 kcal/mol compared to 12.8 and 13.2 for Pathways 2 and 1, respectively, Scheme 1).

**Scheme 2:**
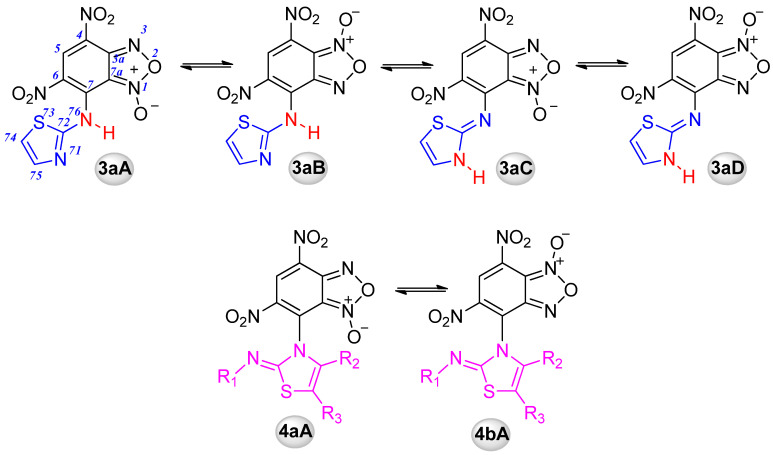
Possible tautomeric forms of benzofuroxans.

Among possible isomers, which can be formed through Pathways 1 and 2 (Scheme 2) both due to the N-1/N-3-oxide equilibrium and/or prototropic tautomerism in the molecule [25,26,27,28], the most stable one is **3aC**; however, the energy difference between models with different locations of hydrogen (at amine/thiazole N: **3aA**/**3aC** and **3aB**/**3aD**) is less than 1 kcal/mole, suggesting the possible coexistence of both structures (Table 1). The preferable location of the benzofuroxan oxygen moiety is at the N1 atom that is almost by 4 kcal/mol more advantageous compared to the tautomers with an oxygen atom located at N3. The products **4aA** and **4aB** that can be obtained by Pathway 2 are unfavorable (Table 1). Thus, one can assume that the reaction will more easily proceed by kinetically close Pathways 1 and 2; however, the first way is preferable, leading to, thermodynamically, much more stable products (**3aC** and **3aA)**.

#### 2.1.2. Synthesis

**Scheme 3:**
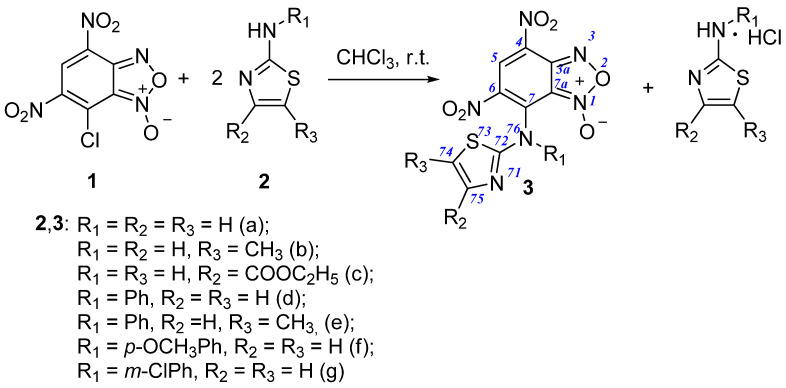
Reaction of 7-chloro-4,6-dinitrobenzofuroxan **1** with 2-aminothiazoles.

The synthesis of benzofuroxan and aminothiazole hybrids (Scheme 3) was realized via S_N_Ar reaction between 7-chloro-4,6-dinitrobenzofuroxan **1** and 2-aminothiazole derivatives **2**, in a 1:2 molar ratio, in chloroform (in CH_2_Cl_2_ for **2c**) at room temperature. The excess of 2-aminothiazole is used to neutralize the hydrogen chloride formed during the reaction.

The complete assignment of the signals in ^1^H, ^13^C and ^15^N NMR spectra was accomplished by using the 2D NMR techniques (COSY, HSQC, HMBC). As already mentioned above, benzofuroxans exist in the form of two tautomers. The presence of only one proton in the structure of the presented compounds significantly complicates the use of two-dimensional NMR experiments. In this case, the chemical shifts of carbons in these tautomeric forms do not differ significantly. Nevertheless, as we showed earlier [25], the difference between C3a and C7a allows us to quite unambiguously assign the observed tautomeric form of the benzofuroxan ring. Although, in the presented samples of compounds we observe only one form, based on a comparison with chemical shifts of previous compounds [25,29], and the establishment of chemical shift C3a is unambiguously 147.3 ppm from a cross-peak in the HMBC (1H-13C) spectrum, allowing us to assign them to the **A** form. The substituents’ position is simply to consider the example of Compound **3d**. In HMBC (^1^H-^15^N), spectra observed cross-peaks from *ortho*-protons of aryl ring (H78) to the N75 on 105 ppm, suggesting the pathway of reaction in Form **3** (see Structure 3 in Scheme 3 for numbering). For other compounds, the assignment can be made by analogy (The sufficiently large spin–spin coupling constants *J* = 1.2 Hz through four bonds observed between methyl protons and H75 in compounds **3b** and **3e** can also be noted. The complete assignment of the spectra (Figures S1–S14, Supplementary Materials) is given in the experimental part.

**Figure 3:**
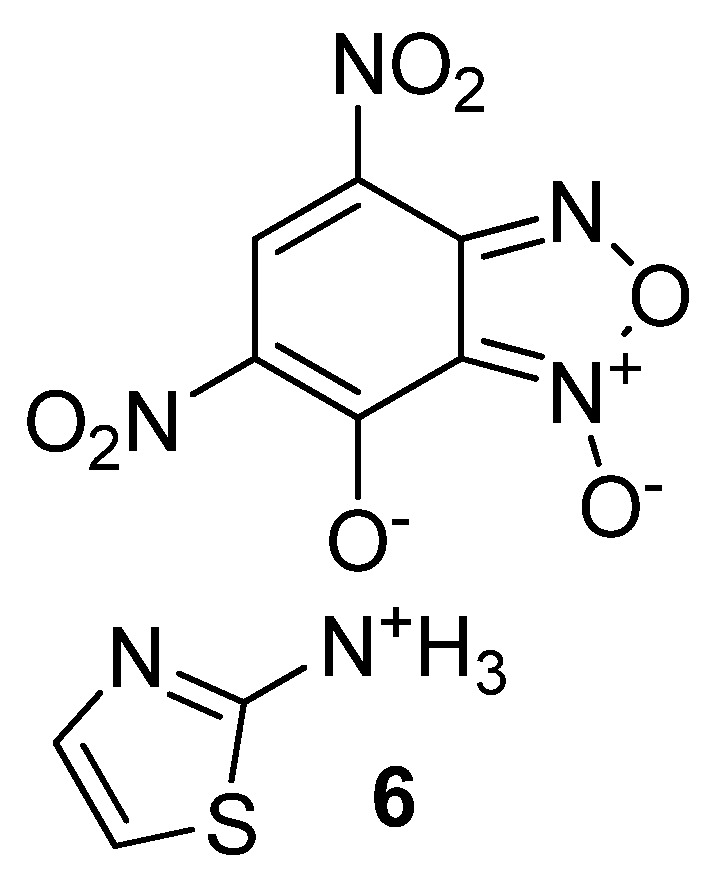
The structure of the salt formed as a by-product.

It should also be noted that, in the formation of Compound **3a**, we observed, in addition to the main reaction product, the formation of a minor product also containing benzofuroxan and aminothiazole fragments; however, the value of the C7 chemical shift (162.7 ppm) did not allow us to relate this compound to any of the tautomers. Column chromatography purification allows for the individual isolation of the major product [20]. Comparison of the signals in ^1^H and ^13^C NMR spectra of mixtures of products with previously obtained results [30] allowed it to be identified as the salt **6** (Figure 3). In the case of the rest of the aminothiazole derivatives, we did not observe the formation of salts as by-products.

**Figure 4:**
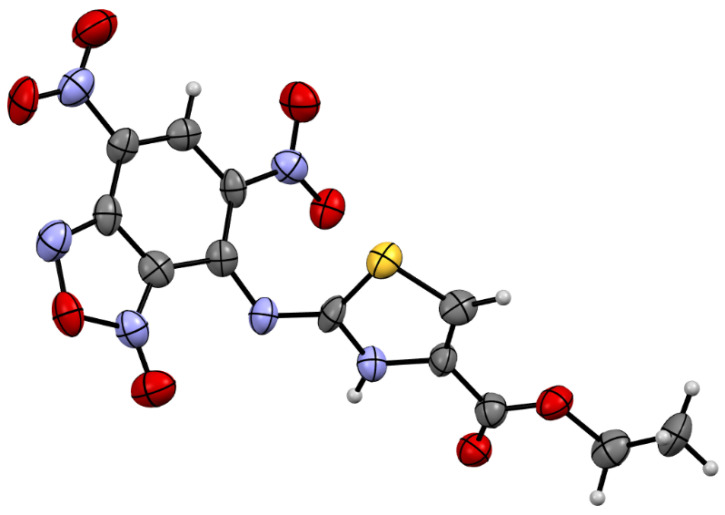
Molecular structure of Compound **3c**. Yellow—sulfur, red—oxygen, blue—nitrogen, and grey—carbon.

According to X-ray data (Figure 4), Compound **3c** crystallizes in the monoclinic space Group *P*2_1_. Benzofuroxan’s bicyclic fragment is planar, and substituted nitro groups deviate from the plane of the bicycle. Hydrogen at the nitrogen atom is placed in geometrically calculated positions in the thiazole ring; however, the XRD data do not allow us to fully answer the question “To which nitrogen atom does hydrogen belong?”.

This result confirms the structure of the product derived from Pathway 1 in Scheme 1.

#### 2.1.3. UV/Vis Spectral Analysis

**Figure 5:**
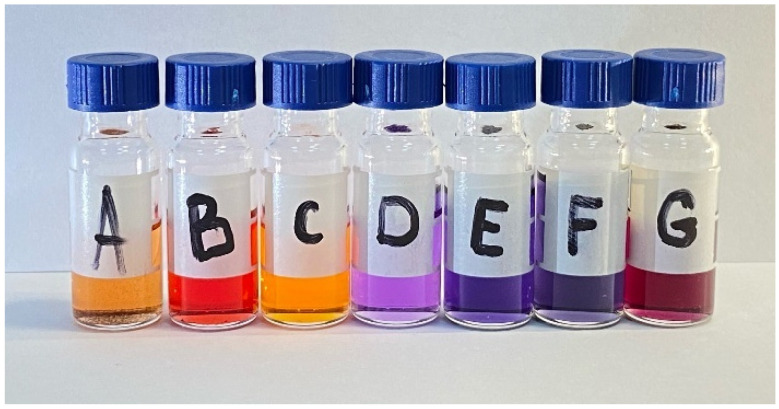
CH_2_Cl_2_ solutions of **3a**–**3g**.

**Figure 6:**
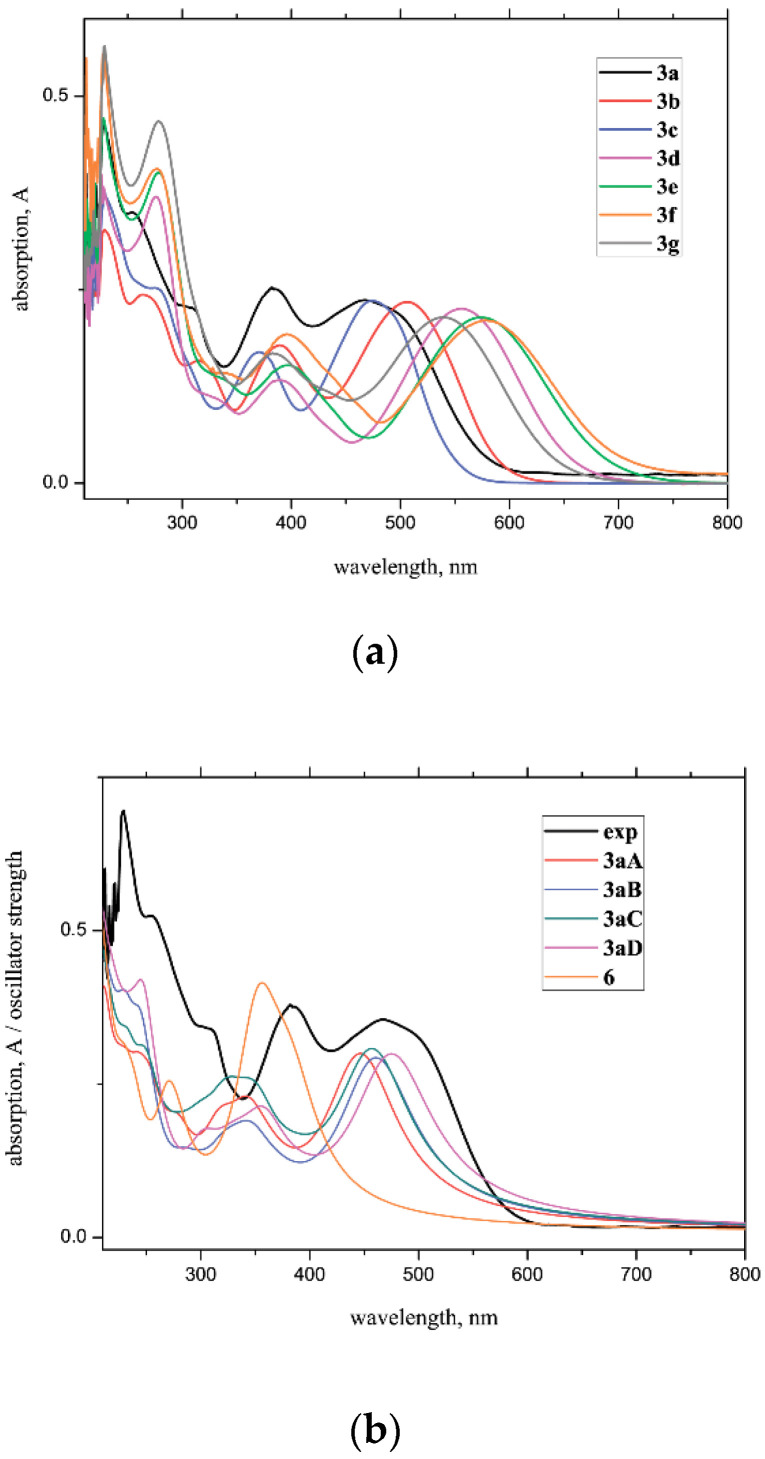
(**a**) Experimental UV/Vis spectra of **3a**–**3g**, in CH_2_Cl_2_; (**b**) experimental UV/Vis spectrum of **3a** and quantum-chemically simulated spectra of **3aA**–**3aD** and **6**.

The nature and position of substituents in **3** significantly affect their color (Figure 5) and their electronic spectra (Figure 6a).

The UV/Vis spectra of **3a**–**3g** demonstrate the lowest energy bands at 465–580 nm depending on R_1_–R_3_ with the shortest wavelength observed for **3a** and **3c** (Figure 6a). It should be mentioned that the lowest energy absorption in the **3a** case consists of two bands. To shed light on the nature of these bands, several possible hypotheses have been considered quantum-chemically: the hydrolysis of **3a** with the formation of **6**, and/or the ‘formal migration’ of the oxygen atom in the benzofuroxan moiety through N-1/N-3-oxide equilibrium and the migration of hydrogen in the aminothiazole moiety by prototropic tautomerism (**3aA**–**3aD**, Scheme 2). The calculations predict a moderate blue shift of the low energy absorption band for Structures B and C compared to A (Figure 6b). Thus, both of them can explain two peaks at 450–500 nm. However, the analysis of relative energies of Isomers **3aA**–**3aD** (Table 1) suggests that the spectrum of **3a** is contributed by **3aA** and **3aC** forms. It should be mentioned that the absorption of hydrolyzed Molecule **6** is predicted to be significantly blue-shifted (by ∼90 nm, Figure 6b) and most probably is associated with the observed experimental band at 383 nm.

The introduction of substituents to both thiazole and amine moieties influences UV/Vis spectra of **3** (Figure 5, Table 2). Thus, in the UV/Vis spectrum of **3b** with a methyl group in the thiazole ring, the longest wavelength is red shifted by ~40 nm compared to **3a**. At the same time, ethoxycarbonyl group (**3c**) almost does not influence the corresponding absorption. Much stronger changes are observed for Compounds **3d**–**3g** with Ph group at amino-group. The replacement of a hydrogen atom by phenyl group leads to a significant red shift of the considered band by 90 nm. Similar to **3a**–**3b**, the introduction of Me into a thiazole fragment results in a red shift of the band from 556 to 573. Additional tuning of absorption wavelength and color of Bf-aminothiazoles can be achieved by substituents in the Ph ring. The OMe group bathochromically shifts the band at 556 to 580 nm; at the same time, the Cl atom leads to an inverse blue shift to 540 nm. The computations correctly predict observed trends (Figure S15, Supplementary Materials; Table 2). According to computations, the longest wavelength absorptions are caused by transitions between the highest occupied and lowest unoccupied molecular orbitals (HOMO-LUMO), which are located at the whole molecules (Figure S16, Supplementary Materials). Nevertheless, for the compounds **3a**–**3c**, the contribution of benzofuroxan moieties both to HOMOs and LUMOs is higher compared to aminothiazole fragments. For the **3d**–**3g** compounds, HOMOs are mostly localized on the Ph-aminothiazole moieties and LUMOs are mostly contributed by benzofuroxan fragments; thus, in these cases, corresponding transitions have a more pronounced charge transfer character, resulting in the higher wavelengths. It should be mentioned that both HOMO and LUMO of hydrolyzed Compound **6** are localized on the benzofuroxan.

### 2.2. Biological Studies

#### 2.2.1. Cytotoxic Assay

Chemotherapy remains one of the most important methods of treating cancer, which, according to statistics, continues to rank second in the list of causes of death after cardiovascular diseases. For an effective fight against malignant tumors, a constant renewal of the drugs used is required. In this regard, the synthesized compounds were screened for cytotoxicity against a number of cancer and normal cell lines.

The cytotoxicity of all compounds against human normal and cancer cell lines was studied at concentrations of 1–100 μM. The IC_50_ data for the tested compounds are presented in Table 3. The drugs tamoxifen and 5-fluorouracil widely used in medical practice were used as reference substances. It is seen that, in relation to the M-HeLa cancer line, the compounds **3a**, **3d**, **3f** exhibit moderate cytotoxicity. Moreover, the Substances **3a**, **3b**, **3e** and **3f** turned out to be more active than their starting compounds. Compounds **3a**, **3d** and **3f** are more active in relation to the M-HeLa cancer line than the reference drug 5-fluorouracil and are slightly inferior to the reference substance tamoxifen. At the same time, the hybrid compounds **3a**, **3d** and **3f** are more selective in relation to normal cells than reference substances, that is, they are not toxic. In relation to the cancer cell lines HuTu 80, PANC-1 and the normal cell line Chang Liver, all tested compounds were found to be non-toxic.

#### 2.2.2. Induction of Apoptotic Effects by Test Compounds

Currently, apoptosis is one of the main mechanisms of cytotoxic activity used in the development of new anticancer drugs [31]. Therefore, it is of considerable interest to study the apoptosis-inducing effect of the lead compound **3f**. The study of apoptotic effects by flow cytometry makes it possible to establish whether the cytotoxic effect of the test compound is associated with the induction of apoptosis in M-HeLa cells.

**Figure 7:**
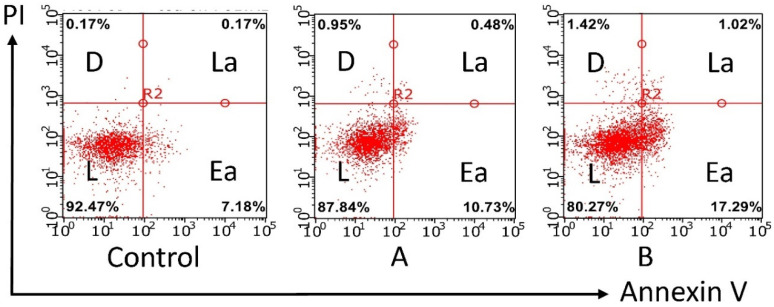
Apoptotic effects of **3f** on M-HeLa cells. (**A**) M-HeLa cells were treated with the indicated compound **3f** at a concentration of 50 µM. (**B**) M-HeLa cells were treated with the indicated compound **3f** at a concentration of 100 µM. The values are presented as the mean ± SD (*n* = 3). L—living cells; D—dead cells; Ea—early apoptotic cells; La—late apoptotic cells.

As shown in Figure 7, after 24 h of incubation of the cells in the presence of Compound **3f** at concentrations of 50 and 100 μM, apoptotic effects were observed. Moreover, they were most pronounced at the stage of early apoptosis at a concentration of 50 μM; Compound **3f** induced early apoptosis in 10.73% M-HeLa cells. With an increase in the concentration of **3f** to 100 μM, the number of apoptotic cells increased to 17.29%. The results suggest that the cytotoxic effect of **3f** in relation to M-HeLa cancer cells can be explained by an apoptotic pathway.

#### 2.2.3. Effects on the Mitochondrial Membrane Potential (Δψm)

Next, we studied the possible mechanism of the apoptosis-inducing effect of the compounds under study on cancer cells. There are two main mechanisms for the induction of apoptosis: an external pathway through death receptors and an internal pathway accompanied by a disruption of the mitochondrial membrane, which leads to a decrease in its potential and is a key indicator of the state of cells [32]. The ability of the studied compounds to cause a decrease in the mitochondrial membrane potential (Δψm) in the cells of the M-HeLa culture was evaluated using the example of the lead compound **3f**. The studies were carried out by flow cytometry methods using the JC-10 reagent. In normal cells with a high mitochondrial membrane potential, the dye JC-10 forms aggregates (J-aggregate) near the mitochondrial membranes. When the membrane potential, due to the stimulation of apoptosis, falls, JC-10 is evenly distributed in the cell as a monomer (J-monomer). JC-10 aggregates in normal cells have red fluorescence, while JC-10 monomers are green.

The ratio between red and green fluorescence can be used to judge the onset of apoptosis.

**Figure 8:**
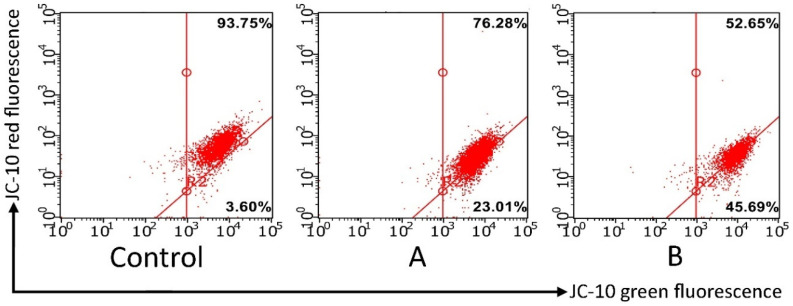
Flow cytometry analysis of M-HeLa cells treated with Compound **3f**, along with the quantification of % of cells with red and green aggregates. (**A**) M-HeLa cells were treated with the indicated compound **3f** at a concentration of 50 µM. (**B**) M-HeLa cells were treated with the indicated compound **3f** at a concentration of 100 µM. The values are presented as mean ± SD.

A reduction in Δψm was demonstrated using flow cytometry analysis (Figure 8). The intensity of the red fluorescence decreased with the increasing concentration of the test compound.

The results obtained suggest that the mechanism of action of the studied compounds may be associated with the induction of apoptosis, which proceeds along the mitochondrial pathway.

#### 2.2.4. Cell Cycle Analysis

**Figure 9:**
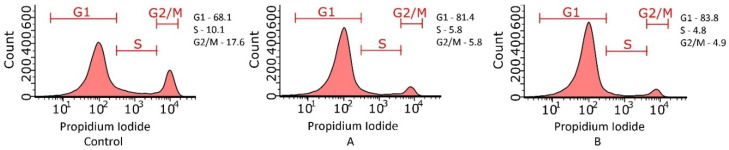
Effect of **3f** on cell cycle progression of M-HeLa by flow cytometry. (**A**) M-HeLa cells were treated with the indicated compound **3f** at a concentration of 50 µM. (**B**) M-HeLa cells were treated with the indicated compound **3f** at a concentration of 100 µM.

Cell cycle analysis by quantification of DNA content is a reliable method to investigate which phase cell cycle has been arrested, wherein propidium iodide dye is used which binds in proportion to the amount of DNA present in the cell [33]. Results of cell cycle analysis using test Compound **3f** against M-HeLa cell lines by flow cytometry showed a transient G1 arrest of cells peaking at 24 h. The results reveal that **3f** caused an increase in % of G1 arrest. Compound **3f** was on 81.4% and 83.8% at 50 and 100 µM, respectively, which is more than control cells (68.1%) (Figure 9).

Regulation of the cell cycle is carried out by the activation of successively replacing cyclin-dependent kinases, which are a holoenzyme complex consisting of the catalytic subunit (Cdk) itself and the regulatory subunit, cyclin. Cyclin binding increases the kinase activity of Cdk and determines their localization and substrate specificity. The expression level of each of the cyclins and Cdk changes directionally at certain phases of the cell cycle. Thus, the transition from the G1 phase to the S phase is associated with the formation of complexes of cyclin E with Cdk2. Based on the literature data [34], it can be assumed that the arrest of the cell cycle at the G1 stage may be due to a decrease in the expression of cyclin E and Cdk2 after the treatment of cells with the leader compound **3f**. The expression of indicated proteins is further planned to be analyzed by Western blot analysis.

Thus, the studies carried out have shown the perspective of introducing aminothiazole fragments into the benzofuroxan molecule. Compounds **3a**, **3d** and **3f** were more active in relation to the M-HeLa cancer line than the reference drug 5-fluorouracil and are slightly inferior to the reference substance tamoxifen. At the same time, Hybrid Compounds **3a**, **3d** and **3f** are more selective in relation to normal cells than reference substances, that is, they are not toxic. The highest activity was shown by Compound **3f** containing methoxyphenyl as an additional substituent in the aminothiazole fragment. The importance of the methoxyphenyl fragment was also previously shown by us in previous studies of the antimicrobial activity of benzofuroxan derivatives [35]. The compound-leader **3f** with the best IC_50_ in relation to the M-HeLa cancer cell line is promising for the production on their basis of low-toxic antitumor agents with selective cytotoxicity and tropism to tumor cells and not showing a cytotoxic effect in relation to normal cells.

#### 2.2.5. Absorption, Distribution, Metabolism, Excretion, and Toxicity Data (ADMET) Predictions

ADMET properties of Starting Compound **1** and Hybrid Compounds **3a–g** were investigated through SwissADME program (http://swissadme.ch/, accessed on 7 July 2021). According to Lipinski’s rule of five [36] and Ghose drug-like filter [37], all the designed compounds were in accordance with the rules by causing no more than one violation; thus, designed compounds seem to possess a good pharmacokinetic profile and might be approved for preclinical trials (Figures S17–S24, Supplementary Materials).

For all compounds, an inhibitory activity of Cytochrome P450 2C19 (CYP2C19) was found, whereas inhibitory activity of CYP2C9, another member of the CYP2C subfamily of the cytochrome P450, was found for Compounds **3d**, **3e**, and **3f**. Inhibitory activity of **3c** and **3f** towards CYP3A4 was also predicted. Always in the case of pharmacokinetics predictions, P-gp was recognized as a possible substrate for **3a**, **3b**, and **3c** (see Supplementary Materials). 

In addition, ADMET predictions for Compounds **1** and **3a**–**f** provided a list of their possible molecular targets (http://www.swisstargetprediction.ch/, accessed on 7 July 2021). It is worth noting that the most active compound, **3f**, has seven protein targets: proteases Matrix metalloproteinase 9 and Matrix metalloproteinase 2; family A G protein-coupled receptors Adenosine A1 and Adenosine A2a; enzyme PI3-kinase p110-gamma subunit; Dual specificity mitogen-activated protein kinase kinase 1 and MAP kinase p38 alpha while not active parent 7-chloro-4,6-dinitrobenzofuroxan **1** has only one target, while for the least active compounds, **3c** and **3e**, according to the predictions of the site, no possible targets were found. 

## 3. Conclusions

A series of novel benzofuroxans derivatives were synthesized through a nucleophilic aromatic substitution reaction of 7-chloro-4,6-dinitrobenzofuroxan with 2-aminothiazoles. The results of the carried-out quantum-chemical computations show that the attack on the electrophilic C-5 carbon atom of the thiazole ring is kinetically disadvantageous; at the same time, reaction through the exocyclic NH_2_ leads to more thermodynamically preferable products compared to those derived from the involvement of the endocyclic nitrogen atom. The complete assignment of the signals in ^1^H, ^13^C and ^15^N NMR spectra by using the 2D NMR techniques (COSY, HSQC, HMBC) and X-ray data confirmed the results of the quantum-chemical study. The nature and position of substituents in compounds significantly affect their color and UV spectra. For the obtained compounds, the anticancer activity and possible mechanism of action on cancer cells were studied. In relation to the M-HeLa cancer line, the substances **3a**, **3b**, **3e** and **3f** turned out to be more active than starting benzofuroxan, **1**, and aminothiazoles, **2**, while in relation to the normal cell line, Chang-Liver-tested compounds were found to be non-toxic. Using flow cytometry methods, we showed that the mechanism of action of the studied compounds may be associated with the induction of apoptosis, which proceeds along the mitochondrial pathway. Thus, these compounds are promising candidates for further development of anticancer agents.

## 4. Materials and Methods

### 4.1. Chemistry

IR spectra were recorded in KBr or as emulsions in vaseline oil (sample concentration 0.25%) on a Bruker Vector-22 spectrometer in the range 400–4000 cm^−1^; given are the most intense absorption bands. Electronic absorption (UV-Vis) spectra were recorded at room temperature on a Perkin-Elmer Lambda 35 spectrometer (PerkinElmer, Inc, Waltham, MA (Massachusetts), USA) using 10 mm quartz cells. Absorption spectra were registered with a scan speed of 480 nm/min, using a spectral width of 1 nm. All samples were prepared as solutions in dichloromethane with the concentrations ~10^−5^ mol L^−1^. Nuclear magnetic resonance (NMR) spectra were recorded on Brucker spectrometers AVANCE*III*-500 (Bruker BioSpin, Rheinstetten, Germany) (500.1 MHz for ^1^H, 125.8 MHz for ^13^C and 50.7 MHz for ^15^N) in acetone-*d*_6_ at 303 K. Chemical shifts were measured in δ (ppm) with reference to the solvent (δ = 2.10 ppm and 30.5 ppm for (CD_3_)_2_CO for ^1^H and ^13^C NMR, respectively). The ^15^N NMR spectra are referenced to external urea and converted to the liquid anhydrous ammonia scale ((NH_2_)_2_C(O) δ(^15^N) = 75 ppm). The pulse programs of the COSY, HSQC and HMBC experiments were taken from the Bruker software library. Elemental analysis was performed on a CHNS-O Elemental Analyser EuroEA3028-HT-OM (EuroVector S.p.A., Milan, Italy) with an accuracy ±0.4% for C, H, Cl, N and S. The melting points were determined in glass capillaries on a Stuart SMP 10 instrument (Keison Products, Chelmsford, UK). The progress of reactions and the purity of products were monitored by TLC on Sorbfil UV-254 plates (Sorbpolimer, Krasnodar, Russia); the chromatograms were developed under UV light.

#### 4.1.1. X-ray Crystallography Data

The data set for the single crystal **3c** was collected on a Rigaku Synergy S instrument (Rigaku Oxford diffraction, Tokyo, Japan) with a HyPix detector and a PhotonJet microfocus X-ray tube using Cu Kα (1.54184 Å) radiation at a low temperature. Images were indexed and integrated using the CrysAlisPro data reduction package. Data were corrected for systematic errors and absorption using the ABSPACK module: numerical absorption correction based on Gaussian integration over a multifaceted crystal model and empirical absorption correction based on spherical harmonics according to the point group symmetry using equivalent reflections. The GRAL module was used for the analysis of systematic absences and space group determination. The structure was solved by direct methods using SHELXT [38] and refined by the full-matrix least-squares on F2 using SHELXL [39]. Non-hydrogen atoms were refined anisotropically. The hydrogen atoms were inserted at the calculated positions and refined as riding atoms. The figures were generated using the Mercury v4.1 [40] program. Crystals were obtained by the slow evaporation method.

CCDC 2083328 (**3c**) contains the supplementary crystallographic data for this paper. These data can be obtained free of charge via www.ccdc.cam.ac.uk/conts/retrieving.html, accessed on 7 July 2021 (or from the Cambridge Crystallographic Data Centre, 12 Union Road, Cambridge CB2 1EZ, UK; fax: (+44) 1223-336-033; or deposit@ccdc.cam.uk).

#### 4.1.2. Quantum-Chemical Computations 

**Scheme 4:**
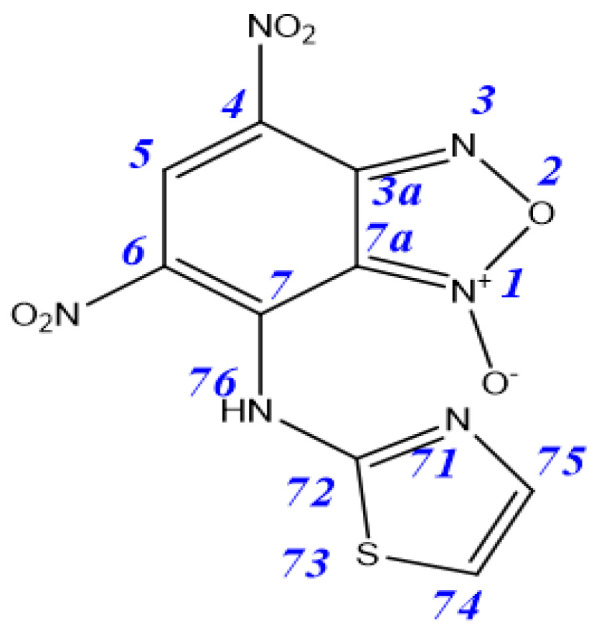
4,6-dinitro-7-(thiazol-2-ylamino)benzo[*c*][1,2,5]oxadiazole 1-oxide (**3a**) (major product).

**Scheme 5:**
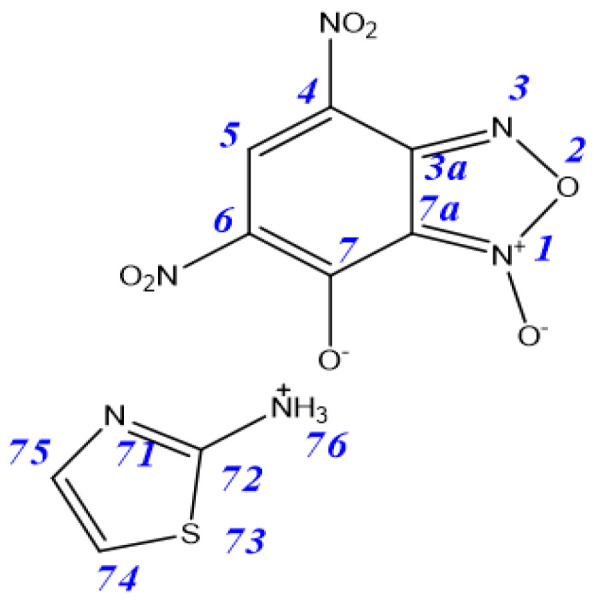
Thiazol-2-aminium 4,6-dinitro-7-oxidobenzo[*c*][1,2,5]oxadiazole 1-oxide (**6**) (minor product).

**Scheme 6:**
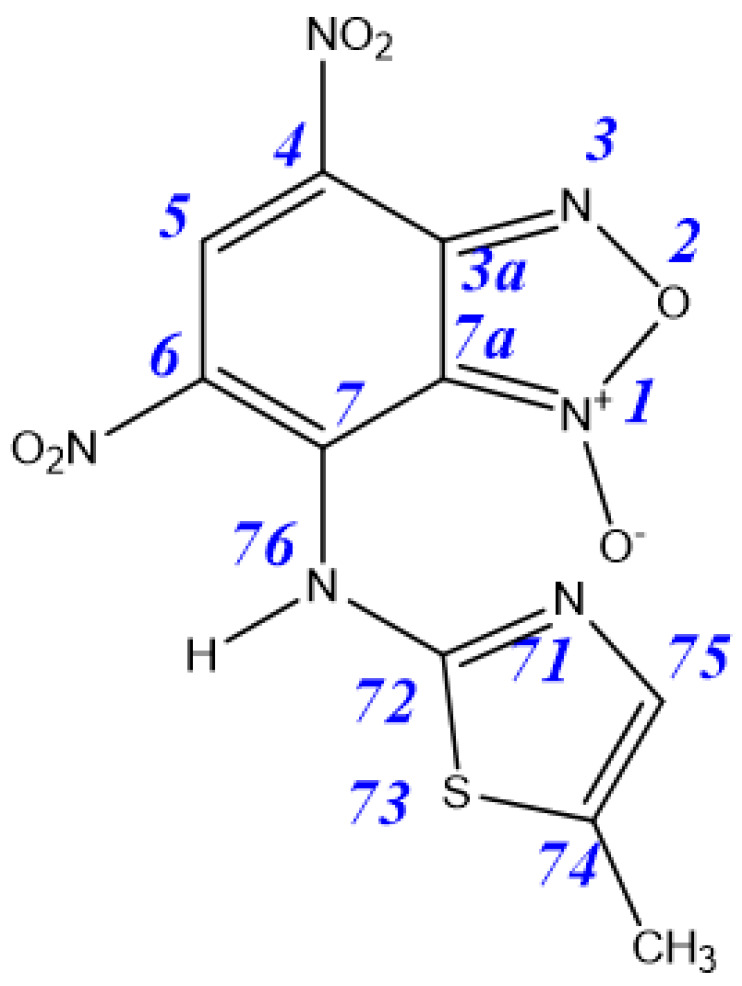
7-(5-methylthiazol-2-ylamino)-4,6-dinitrobenzo[*c*][1,2,5]oxadiazole 1-oxide (**3b**).

**Scheme 7:**
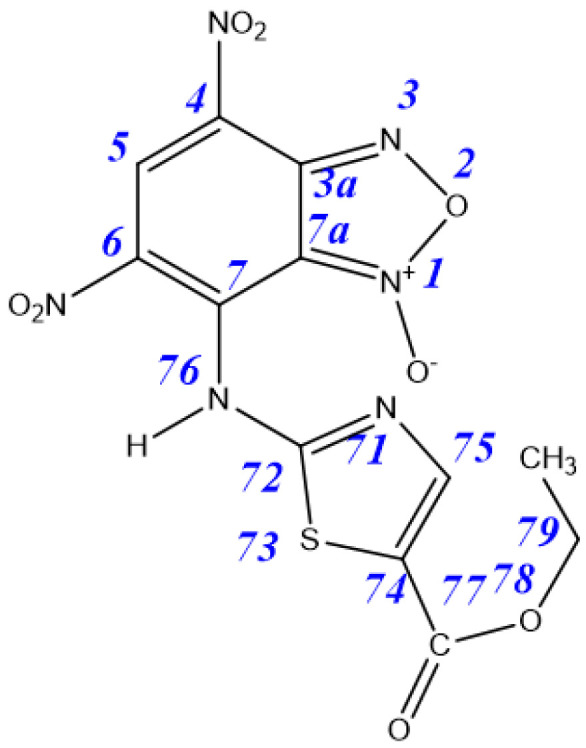
7-(5-(ethoxycarbonyl)thiazol-2-ylamino)-4,6-dinitrobenzo[*c*][1,2,5]oxadiazole 1-oxide (**3c**).

**Scheme 8:**
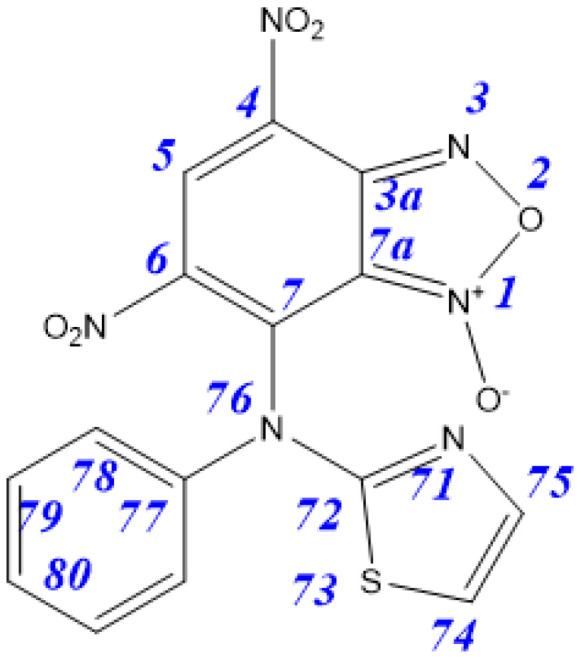
4,6-dinitro-7-(phenyl(thiazol-2-yl)amino)benzo[*c*][1,2,5]oxadiazole 1-oxide (**3d**).

**Scheme 9:**
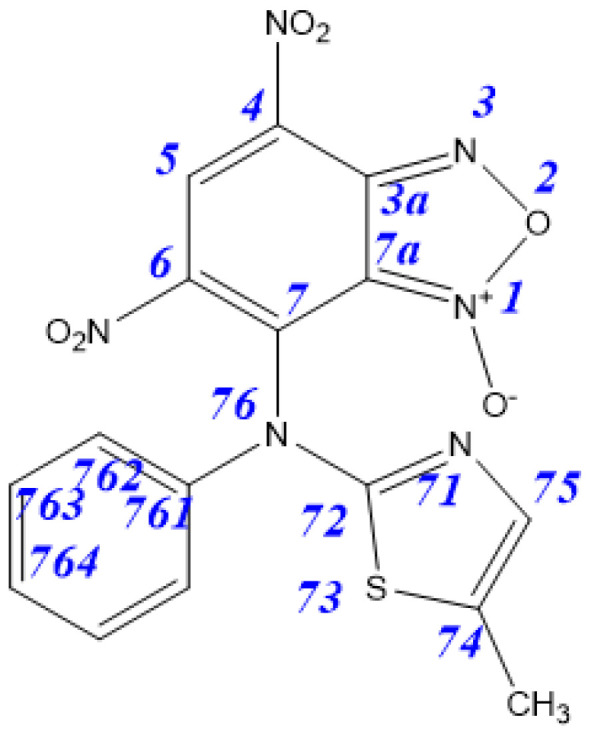
7-((5-methylthiazol-2-yl)(phenyl)amino)-4,6-dinitrobenzo[*c*][1,2,5]oxadiazole 1-oxide (**3e**).

**Scheme 10:**
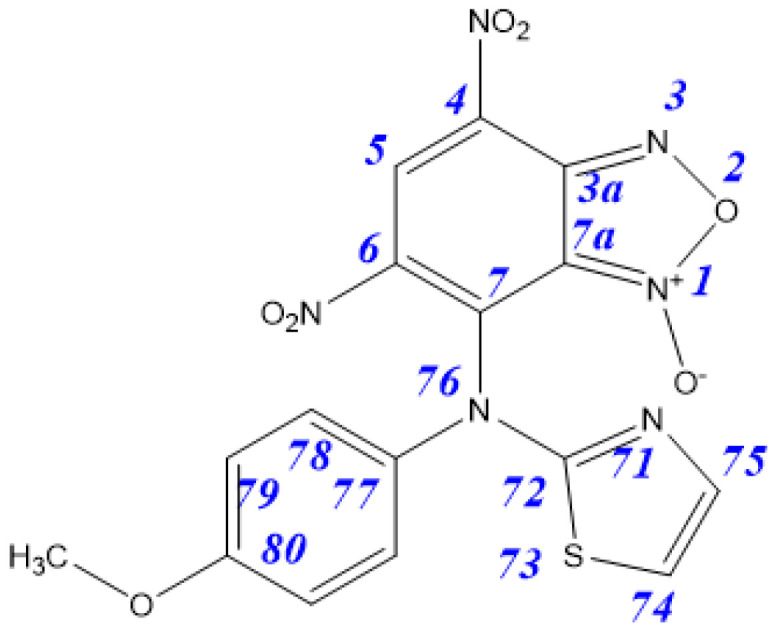
7-((4-methoxyphenyl)(thiazol-2-yl)amino)-4,6-dinitrobenzo[*c*][1,2,5]oxadiazole 1-oxide (**3f**).

**Scheme 11:**
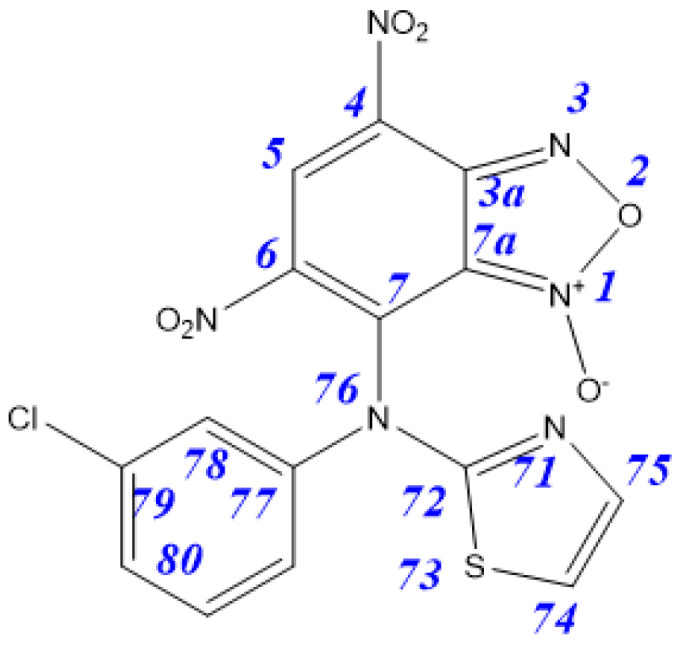
7-((3-chlorophenyl)(thiazol-2-yl)amino)-4,6-dinitrobenzo[*c*][1,2,5]oxadiazole 1-oxide (**3g**).

All quantum-chemical computations were carried out with the use of Gaussian 16 suite of programs [41]. Calculations were performed with Becke’s three-parameter hybrid exchange functional [42] and the gradient-corrected nonlocal correlation functional of Lee et al. [43] (B3LYP) in combination with standard 6–31G* basis set [44,45,46]. For all compounds (Scheme 4, Scheme 5, Scheme 6, Scheme 7, Scheme 8, Scheme 9, Scheme 10 and Scheme 11), geometry optimization of structures was performed without symmetry constraints. To ensure the calculated structures of reagents and products were indeed minima, vibrational analyses were performed using the same methods and were proved by all positive eigenvalues of the Hessian matrix. The transition states were confirmed by the presence of one negative eigenvalue in the Hessian matrix of the second derivatives. All calculations were performed for a singlet surface and the solutions found were tested for stability against perturbations imposed on the wave function using the Stable procedure.

7-Chloro-4,6-dinitrobenzofuroxan **1** was synthesized according to the literature [47], 4,6-dinitro-7-(thiazol-2-ylamino)benzo[*c*][1,2,5]oxadiazole 1-oxide **3a** was prepared according to [20], and 2-aminothiazoles were purchased by Sigma-Aldrich (Darmstadt, Germany).

Reaction between 7-chloro-4,6-dinitrobenzofuroxan **1** and aminothiazoles. A solution of Aminothiazole **2** (0.0016 mol) in 5 mL of CHCl_3_ or CH2Cl2 was added to a solution of 7-chloro-4,6-dinitrobenzofuroxan **1** (0.0008 mol) in 5 mL of CHCl_3_ or CH_2_Cl_2_ at room temperature. The reaction was carried out at room temperature and under magnetic stirring, and the conversion was monitored through TLC analysis (eluent: toluene/ethyl acetate, 2:1). The mixture was stirred at room temperature overnight, the crude mixture was precipitated in hexane (10 mL), and the obtained solid was filtered off, washed with cold water (100 mL), and dried under vacuum (0.06 mm Hg) at 40 °C to constant weight. The crude products were purified by column chromatography (eluent in each case was selected individually) to give the target compounds.

^1^H NMR (500 MHz, Acetone-d_6_) δ 7.30 (d, *J* = 4.4 Hz, 1H, H74), 7.65 (d, *J* = 4.4 Hz, 1H, H75), 9.08 (s, 1H, H5). ^13^C{^1^H} NMR (126 MHz, Acetone-d_6_,) δ 170.1 (C72), 147.2 (C3a), 143.7 (C6), 133.2 (C5), 128.8 (C75), 127.1 (C7), 124.1 (C4), 112.2 (C74), 109.2 (C7a). ^15^N NMR (51 MHz, Acetone-d_6_) δ N6, (N4), (N71), (N76) no observed cross-peaks in spectra.

^1^H NMR (500 MHz, Acetone-d_6_) δ 7.16 (d, *J* = 4.4 Hz, 1H, H74), 7.51 (d, *J* = 4.4 Hz, 1H, H75), 9.24 (s, 1H, H5). ^13^C{^1^H} NMR (126 MHz, Acetone-d_6_) δ 171.6 (C72), 162.7 (C7), 148.6 (C3a), 135.1 (C5), 127.6 (C4), 126.8 (C75), 116.4 (C6), 112.7 (C7a), 109.1 (C74). ^15^N NMR (51 MHz, Acetone-d_6_) δ N6, (N4), (N71), (N76). no observed cross-peaks in spectra.

Eluent: ethyl acetate. Wine-colored powder, yield 62%. M.p.: 210–212 °C. IR (*ν*, cm^–1^): 1376 (NO_2_ symm), 1548 (NO_2_ asymm), 1620 (furoxan ring). ^1^H NMR (500 MHz, Acetone-d_6_) δ 2,44 (d, *J* = 1.2 Hz, 3H, CH3), 7.33 (q, *J* = 1.2 Hz, 1H, H75), 9.04 (s, 1H, H5). ^13^C{^1^H} NMR (126 MHz, Acetone-d*_6_*) δ 171.1 (C72), 148.5 (C3a), 144.4 (C6), 134.5 (C5), 127.7 (C7), 127.3 (C75), 127.3 (C74), 123.4 (C4), 113.9 (C7a), 13.3 (CH_3_). ^15^N NMR (51 MHz, Acetone-d_6_) δ 366.0 (N6), 362,2 (N4), 189.5 (N71), n/o/ (N76). Anal. calcd (%) for C_10_H_6_N_6_O_6_S: C 35.51; H 1.79; N 24.85; S 9.48. Found: C 35.59; H 1.82; N 24.79; S 9.42.

Purified by recrystallization from a mixture of chloroform/hexane (3:1). Purple powder, yield 93%. M.p.: 188–189 °C. IR (*ν*, cm^–1^): 1339 (NO_2_ symm), 1551 (NO_2_ asymm), 1624 (furoxan ring), 1691 (CO), 1712 (C=O), 3101 (C5H). ^1^H NMR (500 MHz, Acetone-d_6_) δ 1.38 (tr, *J* = 7.1 Hz, 3H, CH3), 4.38 (q, *J* = 7.1 Hz, 2H, H79), 8.10 (s, 1H, H75), 9.09 (s, 1H, H5). ^13^C{^1^H} NMR (126 MHz, Acetone-d_6_) δ 165.1 (C72), 160.3 (C77), 148.0 (C3a), 140.0 (C6), 139.0 (C74), 133.0 (C5), 129.9 (C7), 127.5 (C4), 123.9 (C75), 114.0 (C7a), 63.0(C79), 15.1 (C80). ^15^N NMR (51 MHz, Acetone-d_6_) δ 365.2 N6, 361,2 (N4), 223.2 (N71), n/o/(N76). Anal. calcd (%) for C_12_H_8_N_6_O_8_S: C 36.37; H 2.03; N 21.21; S 8.09. Found: C 36.36; H 2.05; N 21.21; S 8.13.

Crystal Data for C_12_H_8_N_6_O_8_S (*M* = 396.30 g/mol): monoclinic, space group P2_1_ (no. 4), *a* = 11.6975(17) Å, *b* = 4.8735(8) Å, *c* = 14.135(3) Å, *β* = 104.348(15)°, *V* = 780.7(2) Å^3^, *Z* = 2, *T* = 99.9(7) K, μ(Cu Kα) = 2.440 mm^−1^, *Dcalc* = 1.686 g/cm^3^, 4524 reflections measured (6.454° ≤ 2Θ ≤ 152.63°), 2377 unique (*R*_int_ = 0.1287, R_sigma_ = 0.1663) which were used in all calculations. The final *R*_1_ was 0.1090 (I > 2σ(I)) and *wR*_2_ was 0.2847 (all data).

Eluent: chloroform. Violet powder, yield 57%. M.p.: 112–113 °C. IR (*ν*, cm^–1^): 1336 (NO_2_ symm), 1556 (NO_2_ asymm), 1621 (furoxan ring). ^1^H NMR (500 MHz, Acetone-d_6_) δ 7.35 (d, *J* = 3.6 Hz, 1H, H74), 7.41 (d, *J* = 3.6 Hz, 1H, H75), 7.49 (d, *J* = 7.6 Hz, 1H, H80), 7.54 (tr, *J* = 7.6 Hz, 2H, H79), 7.63 (d, *J* = 7.6 Hz, 2H, H78), 8.96 (s, 1H, H5). ^13^C{^1^H} NMR (126 MHz, Acetone-d_6_) δ 168.1 (C72), 147.8 (C3a), 143.6 (C77), 141.5 (C75), 140.2 (C6), 135.4 (C7), 134.5 (C4), 131.7 (C5), 131.7 (C79), 130.3 (C80), 126.9 (C78), 117.1 (C74), 115.7 (C7a). ^15^N NMR (51 MHz, Acetone-d_6_) δ 363.8 (N6), 357.5 (N4), 285.1 (N71), 107.3 (N76). Anal. calcd (%) for C_15_H_8_N_6_O_6_S: C 45.00; H 2.01; N 20.99; S 8.01. Found: C 45.05; H 1.98; N 20.95; S 8.06.

Eluent: chloroform. Blue powder, yield 77%. M.p.: 169–170 °C. IR (*ν*, cm^–1^): 1380 (NO_2_ symm), 1557 (NO_2_ asymm), 1621 (furoxan ring). ^1^H NMR (500 MHz, Acetone-d_6_) δ 2.46 (d, *J* = 1.3 Hz, 3H, CH3), 7.08 (q, *J* = 1.3 Hz, 1H, H75), 7.46 (tr, *J* = 8.2 Hz, 1H, H80), 7.52 (tr, *J* = 8.2 Hz, 2H, H79), 7.59 (d, *J* = 8.2 Hz, 2H, H78), 8.94 (s, 1H, H5). ^13^C{^1^H} NMR (126 MHz, Acetone-d_6_) δ 164.8 (C72), 146.8 (C3a), 142.5 (C77), 138.8 (C6), 137.8 (C75), 134.3 (C7), 133.0 (C4), 130.9 (C74), 130.6 (C5), 130.4 (C79), 129.1 (C80), 125.7 (C78), 114.7 (C7a), 11.6 (CH3). ^15^N NMR (51 MHz, Acetone-d_6_) δ 373.3 (N3), 366.1 (N6), 357.1 (N4), 287.0 (N71), 108.5 (N76). Anal. calcd (%) for C_16_H_10_N_6_O_6_S: C 46.38; H 2.43; N 20.28; S 7.74. Found: C 46.31; H 2.49; N 20.33; S 7.68.

Eluent: chloroform. Blue powder, yield 51%. M.p.: 176–178 °C. IR (*ν*, cm^–1^): 1378 (NO_2_ symm), 1558 (NO_2_ asymm), 1621 (furoxan ring), 3101 (C5H). ^1^H NMR (500 MHz, Acetone-d_6_) δ 3.90 (s, 3H, CH3), 7.07 (d, *J* = 9.0 Hz, 2H, H79), 7.32 (d, *J* = 3.5 Hz, 1H, H74), 7.39 (d, *J* = 3.5 Hz, 1H, H75), 7.56 (d, *J* = 9.0 Hz, 1H, H78), 8.94 (s, 1H, H5). ^13^C{^1^H} NMR (126 MHz, Acetone-d_6_) δ 168.8 (C72), 161.6 (C80), 147.8 (C3a), 141.6 (C75), 139.6 (C6), 136.1 (C77), 135.3 (C7), 133.9 (C4), 131.6 (C5), 129.1 (C79), 117.0 (C74), 116.7 (C79), 115.7 (C7a), 56.7 (CH3). ^15^N NMR (51 MHz, Acetone-d_6_) δ 364.2 (N6), 358.6 (N4), 281.2 (N71), 106.4 (N76). Anal. calcd (%) for C_16_H_10_N_6_O_7_S: C 44.65; H 2.34; N 19.53; S 7.45. Found: C 44.62; H 2.37; N 19.49; S 7.49.

Eluent: chloroform. Wine-colored powder, yield 63%. M.p.: 74–75 °C. IR (*ν*, cm^–1^): 1360 (NO_2_ symm), 1556 (NO_2_ asymm), 1621 (furoxan ring). ^1^H NMR (500 MHz, Acetone-d_6_) δ 7.350 (d, *J* = 3.6 Hz, 1H, H74), 7.409 (d, *J* = 3.6 Hz, 1H, H75), 7.485 (d, *J* = 7.6 Hz, 1H, H80), 7.537 (tr, *J* = 7.6 Hz, 2H, H79), 7.626 (d, *J* = 7.6 Hz, 1H, H78), 8.961 (s, 1H, H5). ^13^C{^1^H} NMR (126 MHz Acetone-d_6_) δ 167.4 (C72), 147.7 (C3a), 144.6 (C79), 141.5 (C75), 140.1 (C6), 136.4 (C77), 134.9 (C7), 134.9 (C4), 132.8 (C81), 131.3 (C5), 130.0 (C80), 126.3 (C78), 125.3 (C82), 117.4 (C74), 115.7 (C7a). ^15^N NMR (51 MHz, Acetone-d_6_) δ 373.6 N3, 363.6 N6, 357.5 (N4), 285.4 (N71), 104.7 (N76). Anal. calcd (%) for C_15_H_7_ClN_6_O_6_S: C 41.44; H 1.62; Cl 8.15; N 19.33; S 7.38. Found: C 41.52; H 1.55; Cl 8.23; N 19.37; S 7.44.

### 4.2. Biological Studies

#### 4.2.1. Cytotoxicity Assay

Cytotoxic effects of the test compounds on human cancer and normal cells were estimated by means of the multifunctional Cytell Cell Imaging system (GE Health Care Life Science, Danderyd, Sweden) using the Cell Viability Bio App which precisely counts the number of cells and evaluates their viability from fluorescence intensity data [48]. DAPI and propidium iodide were purchased from Sigma. Two fluorescent dyes that selectively penetrate the cell membranes and fluoresce at different wavelengths were used in the experiments. The M-HeLa clone 11 human, epithelioid cervical carcinoma, the strain of HeLa, a clone of M-HeLa; PANC-1 is a human pancreatic cancer cell line; human duodenal cancer cell line (HuTu 80) from the Type Culture Collection of the Institute of Cytology (Russian Academy of Sciences, S-Petersburg, Russia) and Chang liver cell line (human liver cells) from N. F. Gamaleya Research Center of Epidemiology and Microbiology (Moscow, Russia) were used in the experiments. The cells were cultured in a standard Eagle’s nutrient medium manufactured at the Chumakov Institute of Poliomyelitis and Virus Encephalitis (PanEco company, Moscow, Russia) and supplemented with 10% fetal calf serum and 1% nonessential amino acids. The cells were plated into a 96-well plate (Nunc) at a concentration of 1 × 10^5^ cells/mL, 150 μL of medium per well, and cultured in a CO_2_ incubator at 37 °C. Twenty-four hours after seeding the cells into wells, the compound under study was added at a preset dilution, 150 μL to each well. The dilutions of the compounds were prepared immediately in nutrient media; 5% DMSO that does not induce the inhibition of cells at this concentration was added for better solubility. The experiments were repeated three times. Intact cells cultured in parallel with experimental cells were used as a control.

#### 4.2.2. Flow Cytometry Assay

Cell Culture. M-HeLa cells at 1 × 10^6^ cells/well in a final volume of 2 mL were seeded into 6-well plates. After 24 h of incubation, various concentrations of Compounds **2f** and **3f** were added to wells.

Cell Apoptosis Analysis. The cells were harvested at 2000 rpm for 5 min and, then, washed twice with ice-cold PBS, followed by resuspension in binding buffer. Next, the samples were incubated with 5 μL of annexin V-FITC and 5 μL of propidium iodide for 15 min at room temperature in the dark. Finally, the cells were analyzed by flow cytometry (Guava easy Cyte, MERCK, Kenilworth Union County, NJ, USA). The experiments were repeated three times.

#### 4.2.3. Mitochondrial Membrane Potential

The cells were harvested at 2000 rpm for 5 min and, then, washed twice with ice-cold PBS, followed by resuspension in JC-10 (10 µg/mL) and incubation at 37 °C for 10 min. After the cells were rinsed three times and suspended in PBS, the JC-10 fluorescence was observed by flow cytometry.

#### 4.2.4. Cell Cycle Analysis

The DNA content and cell-cycle distribution after genistein treatment were estimated by flow cytometry. Cell seeding, drug treatment and ethanol fixation were similar to cell proliferation assay. After washing with PBS, genistein and daidzein-treated and -fixed cells were suspended in 150 μL of PBS, then 0.5 mL phosphate-citrate buffer (0.05 M, pH 4.0) was added and the suspension was incubated at room temperature for 5 min to facilitate the extraction of low-molecular-weight DNA. Following centrifugation, the cells were resuspended in 150 μL DNA staining solution (20 μg/mL propidium iodide, 200 μg/mL DNase (RNase-free), and 0.1% Triton X-100) and incubated in the CO_2_ incubator (37 °C for 30 min). Cell cycle distribution was determined by fluorescence-activated cell sorting analysis of propidium iodide-stained ethanol-fixed cells using a Guava EasyCyte (Guava easy Cyte, MERCK, Kenilworth Union County, NJ, USA) [33].

#### 4.2.5. Statistical Analysis

The cytometric results were analyzed by the Cytell Cell Imaging multifunctional system using the Cell Viability BioApp and Apoptosis BioApp application. The data in tables and graphs are given as the mean ± standard error.

#### 4.2.6. ADMET Predictions

The ADMET prediction server used was SwissADME (http://swissadme.ch/, accessed on 7 July 2021) from the Swiss Institute of Bioinformatics.

## Figures and Tables

**Table 1:** Predicted relative energies (kcal/mol) of possible isomers of benzofuroxan and aminothiazole hybrids.

Tautomer	3aA	3aB	3aC	3aD	4aA	4aB
ΔE	0.9	4.6	0	3.9	14.6	16.3

**Table 2:** The experimental and calculated longest wavelength in the UV/Vis spectra of **3a**–**3g**.

	3a	3b	3c	3d	3e	3f	3g
Experiment	467/494	507	475	556	574	581	540
Computations	457	461	456	558	570	597	549

**Table 3:** Cytotoxic effects (IC_50_ μM) of test compounds on the cancer and normal human cell lines *.

Test Compound	Cancer Cell Line			Normal Cell Line
	M-HeLa	HuTu 80	PANC-1	Chang Liver
**1**	94.1 ± 8.6	>100	>100	>100
**2a**	91 ± 8.3	>100	>100	>100
**3a**	61.0 ± 5.4	>100	>100	>100
**2b**	92.0 ± 7.9	>100	>100	>100
**3b**	70.0 ± 6.2	>100	>100	>100
**2c**	63.3 ± 5.7	>100	>100	95.4 ± 8.7
**3c**	95.8 ± 8.4	>100	>100	100 ± 9.4
**3d**	61.0 ± 5.6	>100	>100	86.1 ± 7.8
**2e**	92 ± 8.2	>100	>100	>100
**3e**	79 ± 6.3	>100	>100	85.8 ±7.4
**2f**	93.8 ± 8.4	>100	>100	>100
**3f**	56.6 ± 4.7	>100	>100	>100
Tamoxifen	28.0 ± 2.5	-	-	46.2 ± 3.5
5-Fluorouracil	62.0 ± 4.9	65.2 ± 5.5	68.3 ± 5.7	72.4 ± 6.5

* The experiments were repeated three times. The results are expressed as the mean ± standard deviation (SD); - no cytotoxic effect.

## Data Availability

The data presented in this study are contained within the article or in Supplementary Materials, or are available on request from the corresponding author Elena Chugunova.

## Supplementary Materials

The following are available online at https://www.mdpi.com/article/10.3390/ijms22147497/s1: Figures S1–S14 copies of NMR spectra of all synthesized compounds, Figure S15–Experimental (black) vs. theoretical (red) UV/Vis spectra of **3a**–**3g**, S16–Frontier orbitals of **3a**–**3g** and **6**, Figure S17–S24 ADMET predictions.
