# Supplementary material for: Novel Hybrid Compounds Containing Benzofuroxan and Aminothiazole Scaffolds: Synthesis and Evaluation of Their Anticancer Activity
Source: Int J Mol Sci. 2021 Jul 13;22(14):7497. doi: 10.3390/ijms22147497 (PMC8305974; doi:10.3390/ijms22147497)
Supplement: Supplementary file 1 [file ijms-22-07497-s001.zip › ijms-1288443-supplementary.pdf]

SUPPLEMENTARY MATERIALS

Novel hybrid compounds containing benzofuroxan and aminothiazole scaffolds: synthesis and evaluation of their anticancer activity.

Elena Chugunova [1,2,*], Gabriele Micheletti [3], Dario Telese [3], Carla Boga [3], Daut Islamov [1], Konstantin Usachev [5], Alexander Burilov [1,2], Alena Tulesinova [4], Alexandra Voloshina [1], Anna Lyubina [1], Syumbelya Amerhanova [1], Tatiana Gerasimova [1], Aisylu Gilfanova [1], Victor Syakaev [1]

[1] Arbuzov Institute of Organic and Physical Chemistry, FRC Kazan Scientific Center, Russian Academy of Sciences, Akad. Arbuzov st. 8, Kazan, Tatarstan 420088, Russia;
[2] Laboratory of Plant Infectious Diseases, FRC Kazan Scientific Center of Russian Academy of Sciences, Lobachevskogo st. 2/31, Kazan, Tatarstan 420111 Russia.
[3] Department of Industrial Chemistry 'Toso Montanari', Alma Mater Studiorum –Universita di Bologna Viale Del Risorgimento, 4 Bologna, 40136, Italy.
[4] The Kazan National Research Technological University, Karl Marx st., 68, Kazan, 420015, Russia.
[5] Kazan Institute of Biochemistry and Biophysics, FRC Kazan Scientific Center of Russian Academy of Sciences, Institute of Chemical Engineering and TechnologyLobachevskogo st. 2/31, Kazan, Tatarstan 420111 Russia;
* Correspondence: chugunova.e.a@gmail.com (E.C.); gabriele.micheletti3@unibo.it (G.M.), carla.boga@unibo.it (C.B.); Tel.: +7-843-272-7324 (E.C.); Tel.: +39-051-209-3616 (C.B.; G.M.)

Contents

Copies of NMR spectra 2-15
UV/Vis spectral studies 16-17
ADMET predictions....................18-25

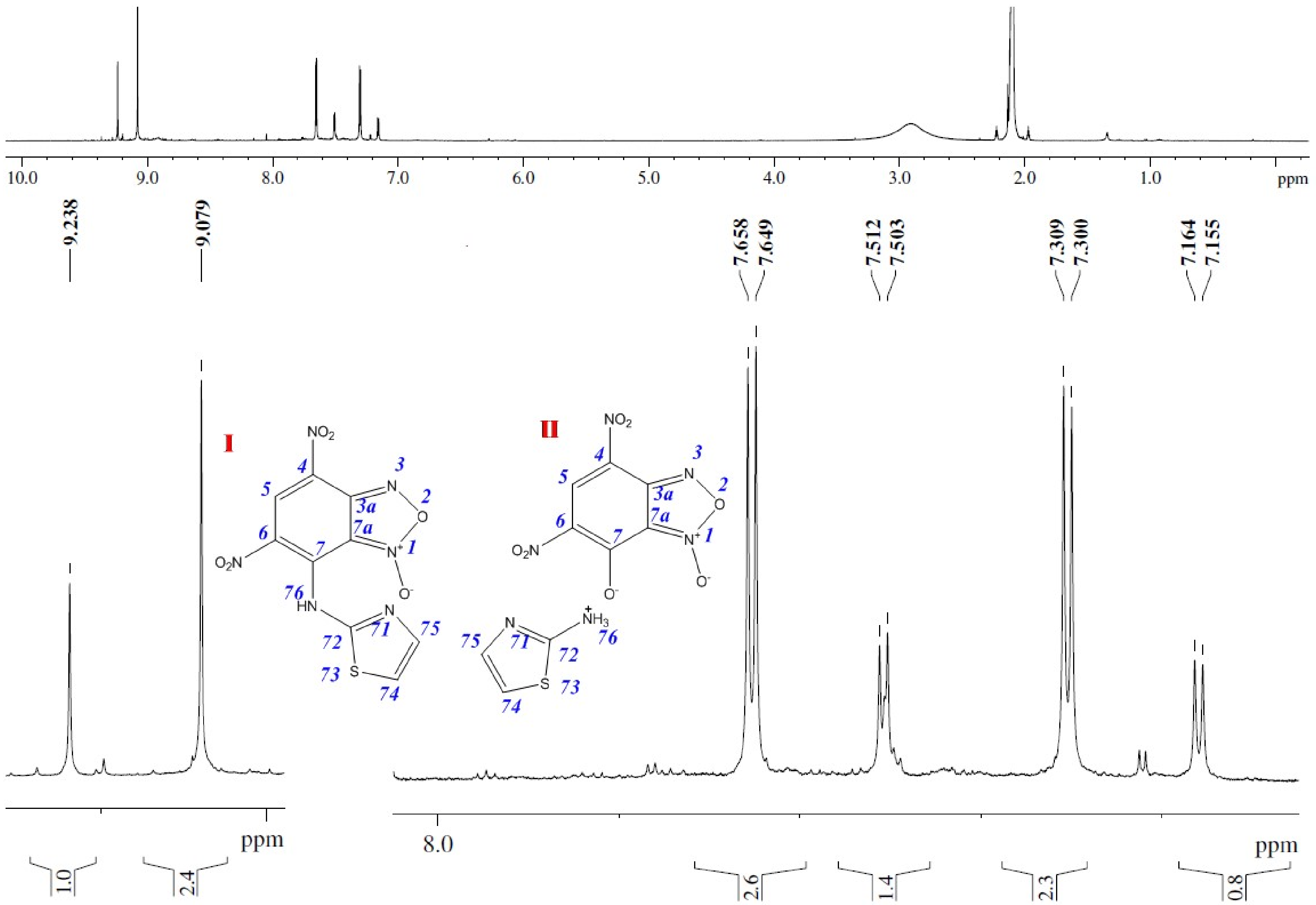

Figure S1. ^{1}H NMR (acetone-d_6, 500 MHz, 303 K) mixture of compound **3b** and **6**.

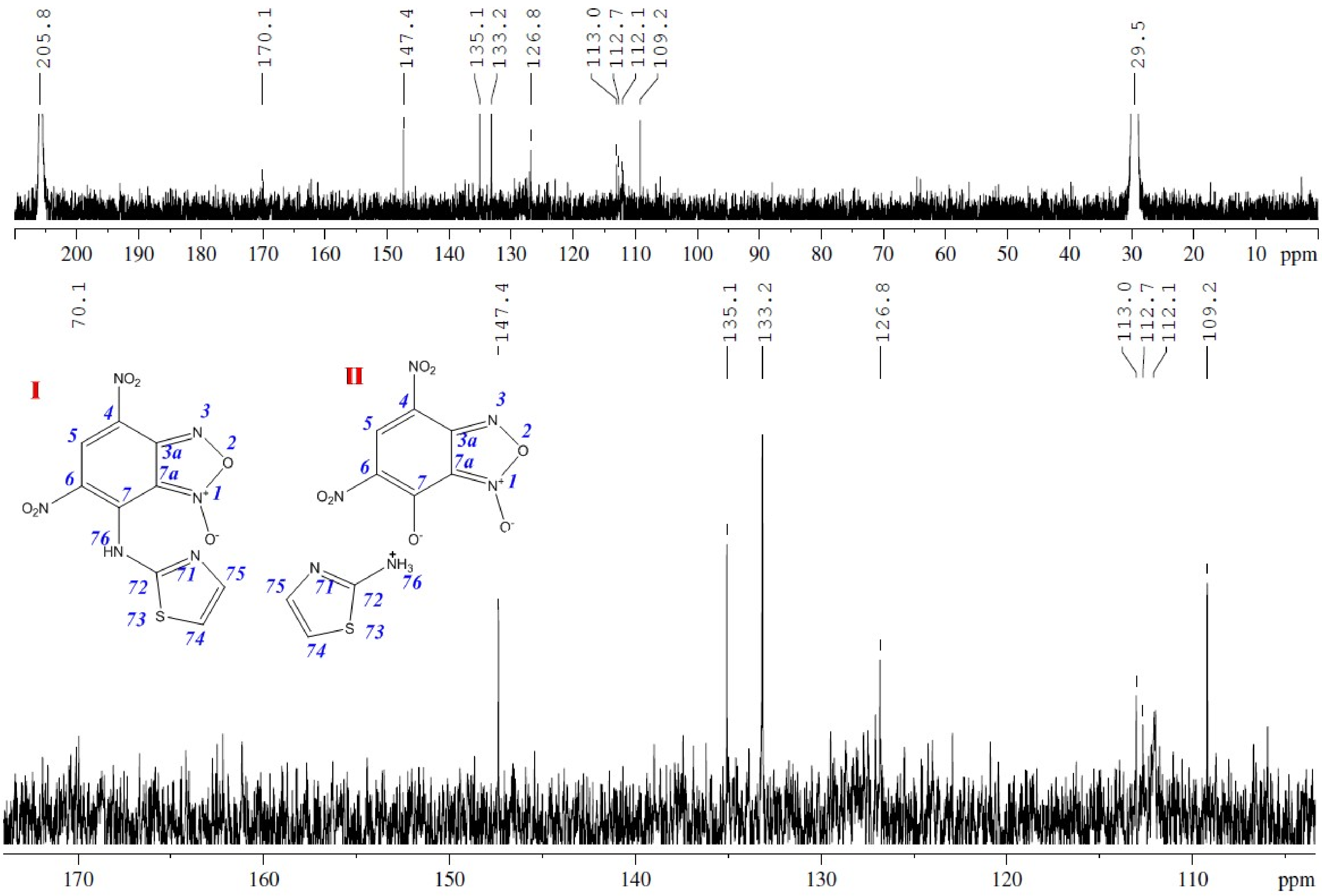

Figure S2. $^{13}C\{1H\}$ NMR (acetone-d_6, 126 MHz, 303 K) mixture of compound **3b** and **6**.

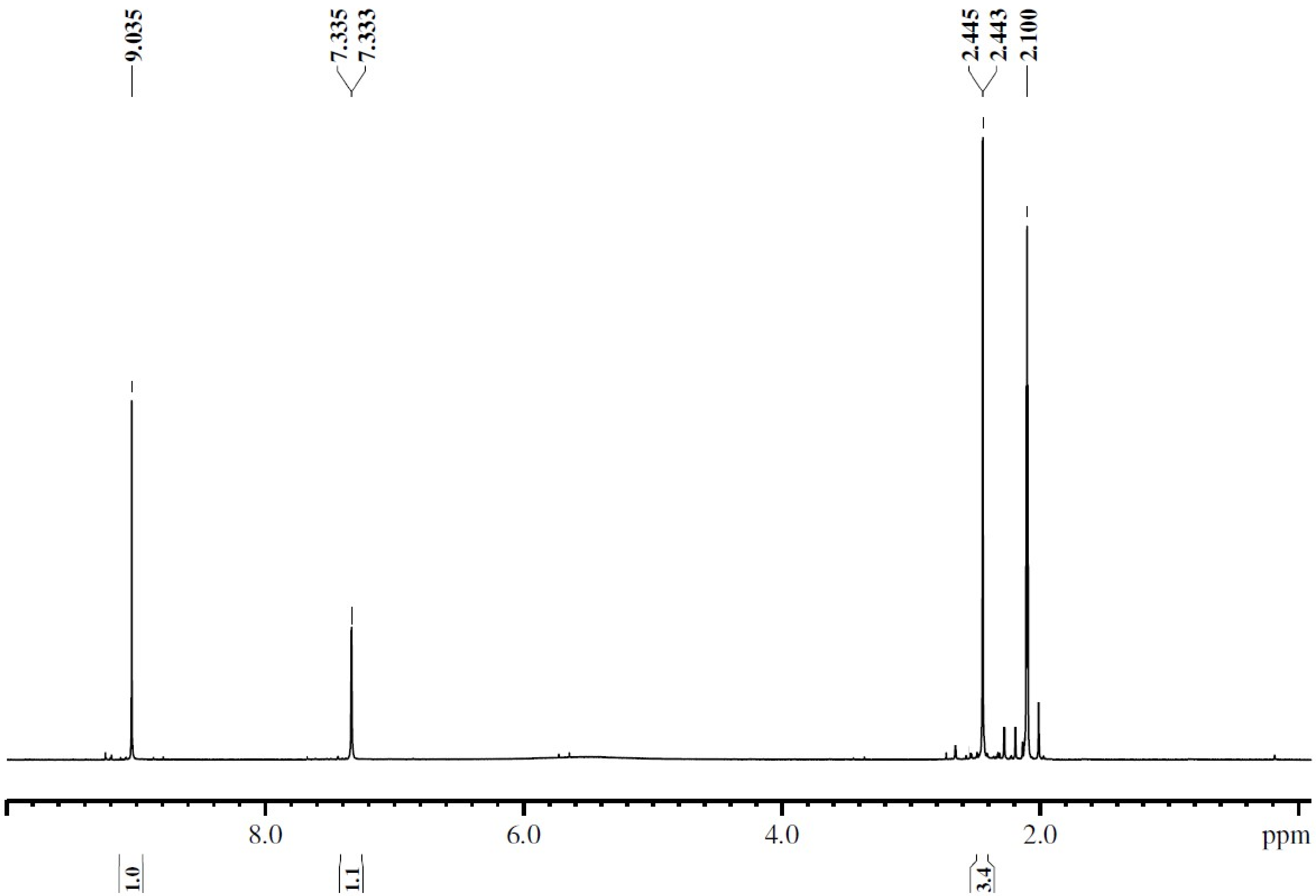

Figure S3. ^{1}H NMR (acetone-d_6, 500 MHz, 303 K) of compound **3b**.

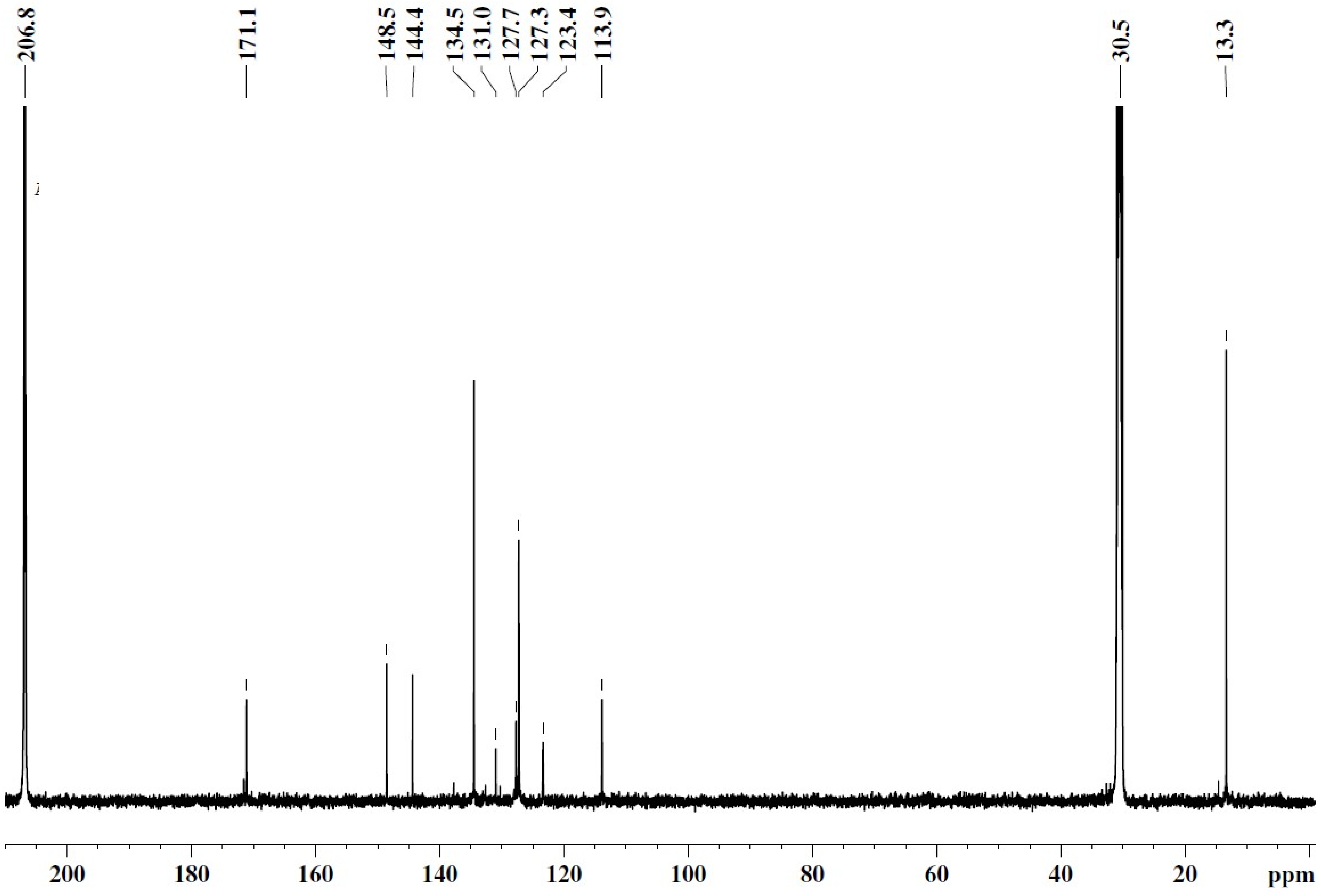

Figure S4. $^{13}C\{1H\}$ NMR (acetone-d_6, 126 MHz, 303 K) of compound **3b**.

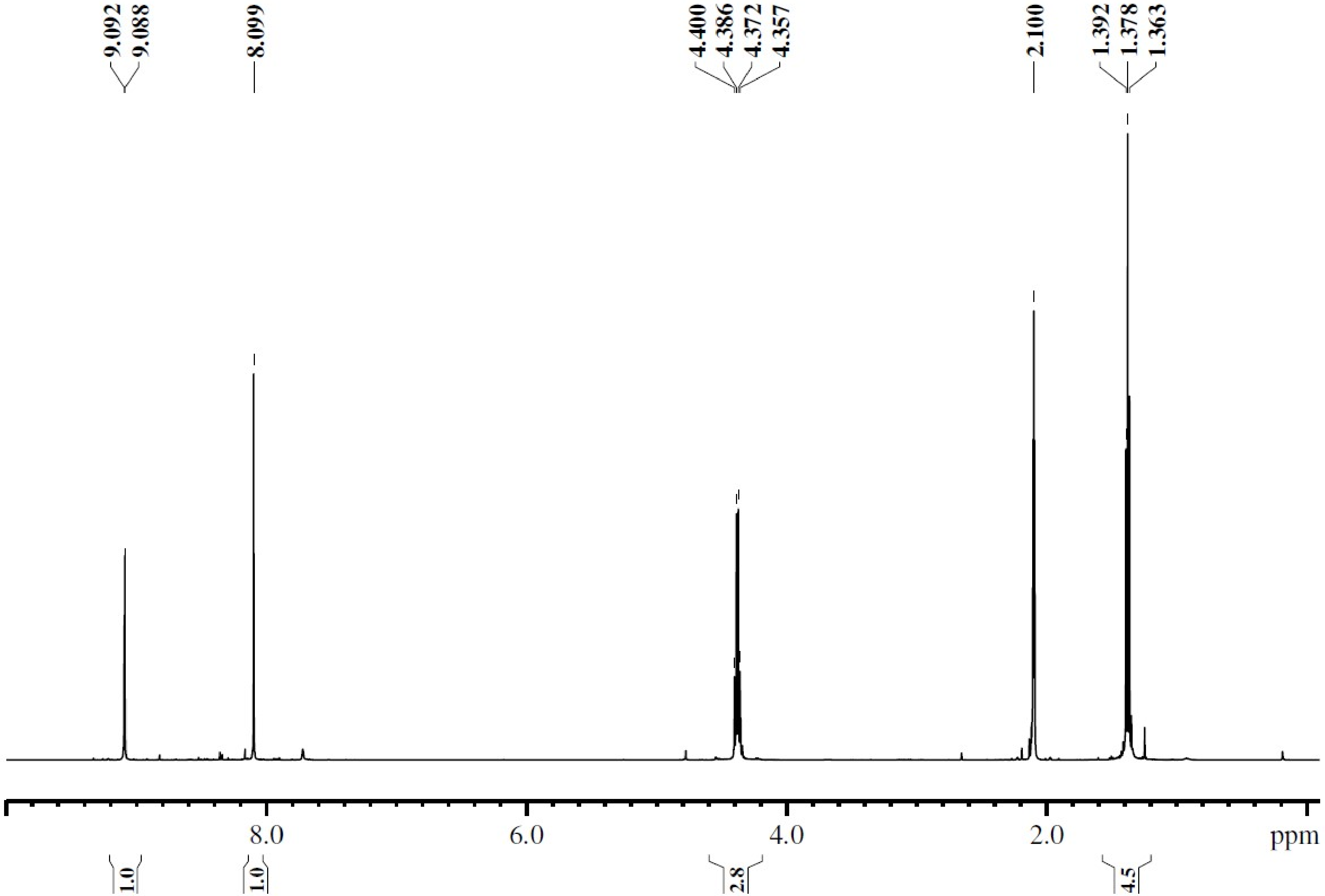

Figure S5. ^{1}H NMR (acetone-d_6, 500 MHz, 303 K) of compound **3c**.

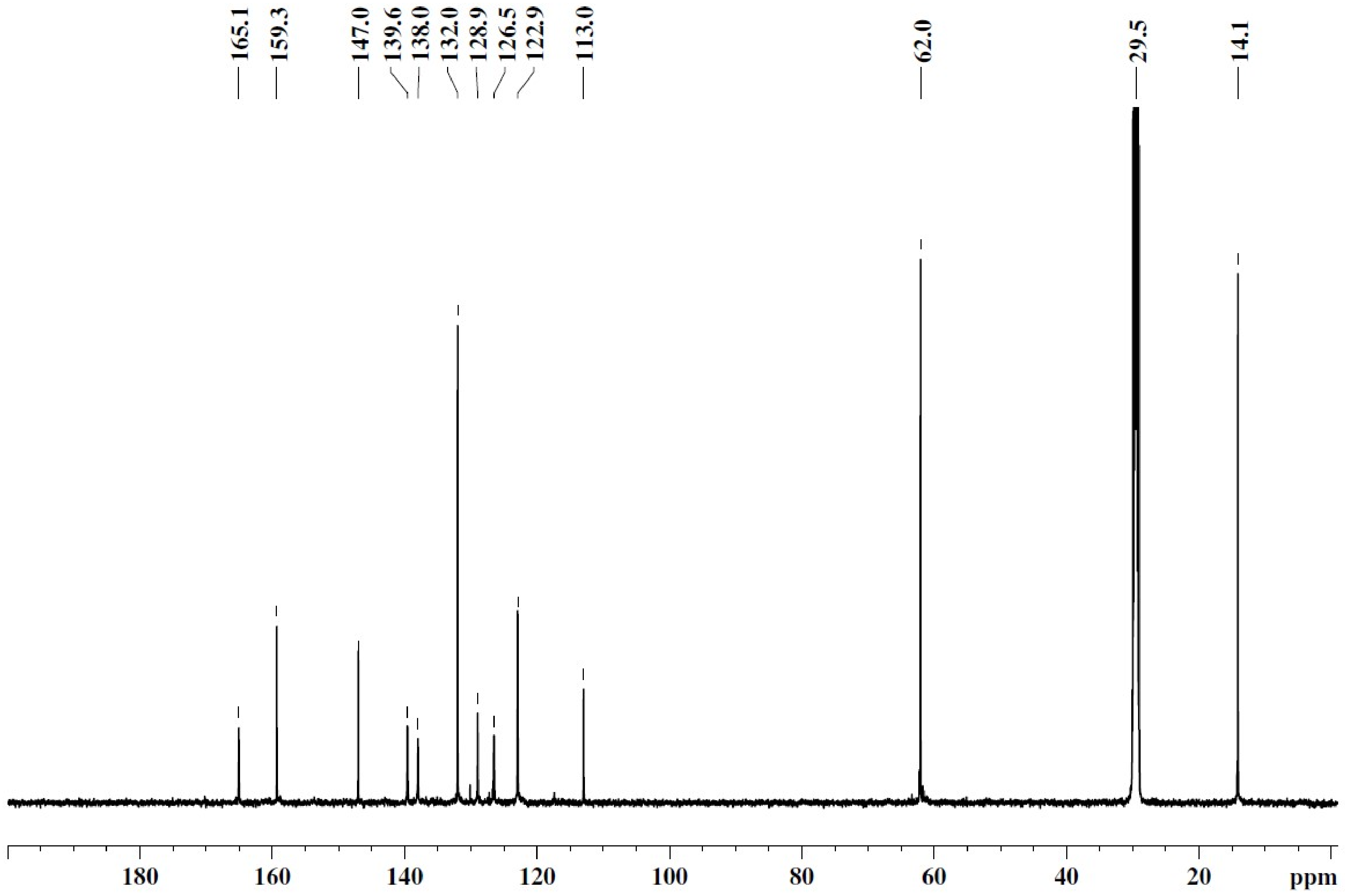

Figure S6. $^{13}C\{1H\}$ NMR (acetone-d_6, 126 MHz, 303 K) of compound **3c**.

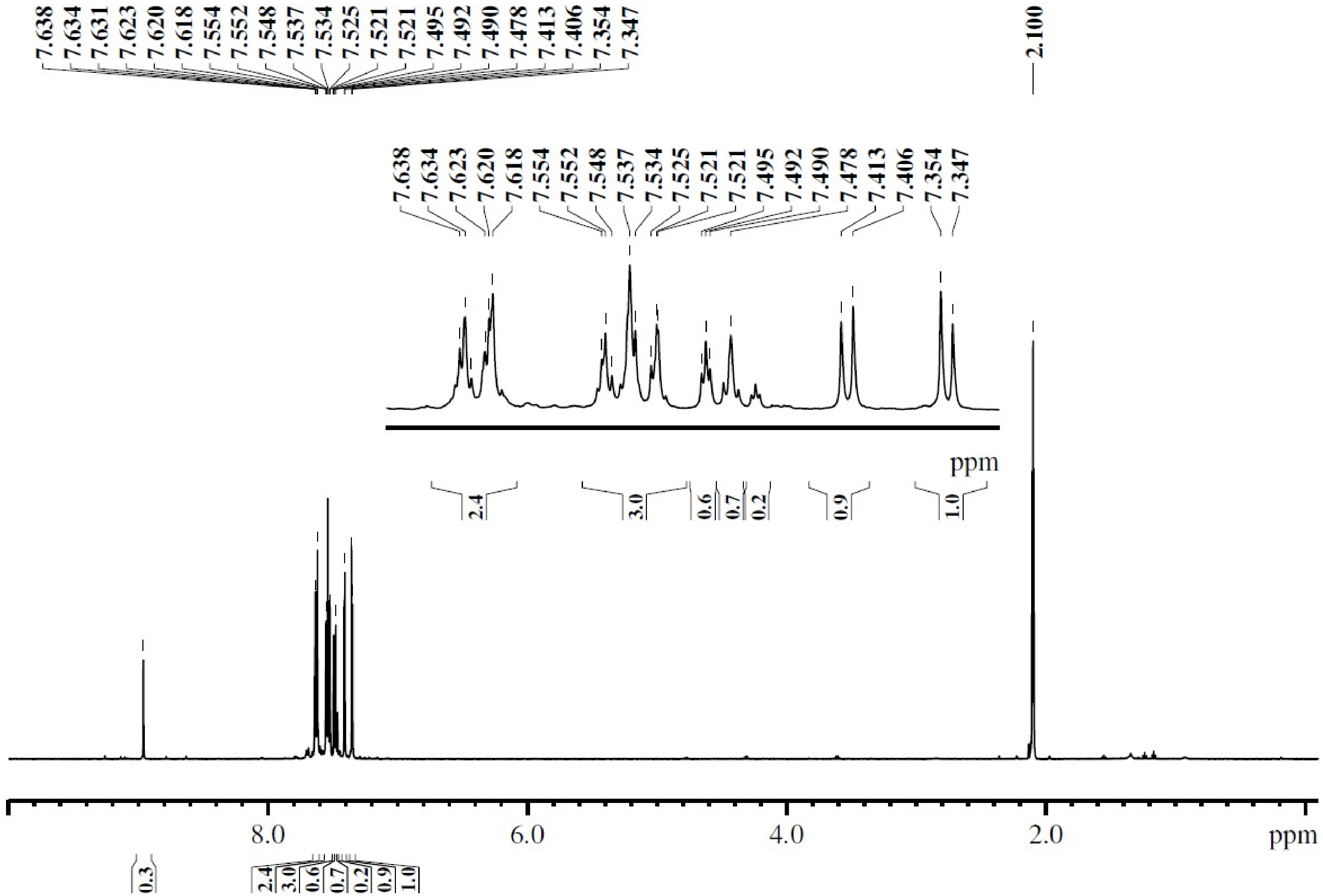

Figure S7. ^{1}H NMR (acetone-d_6, 500 MHz, 303 K) of compound **3d**.

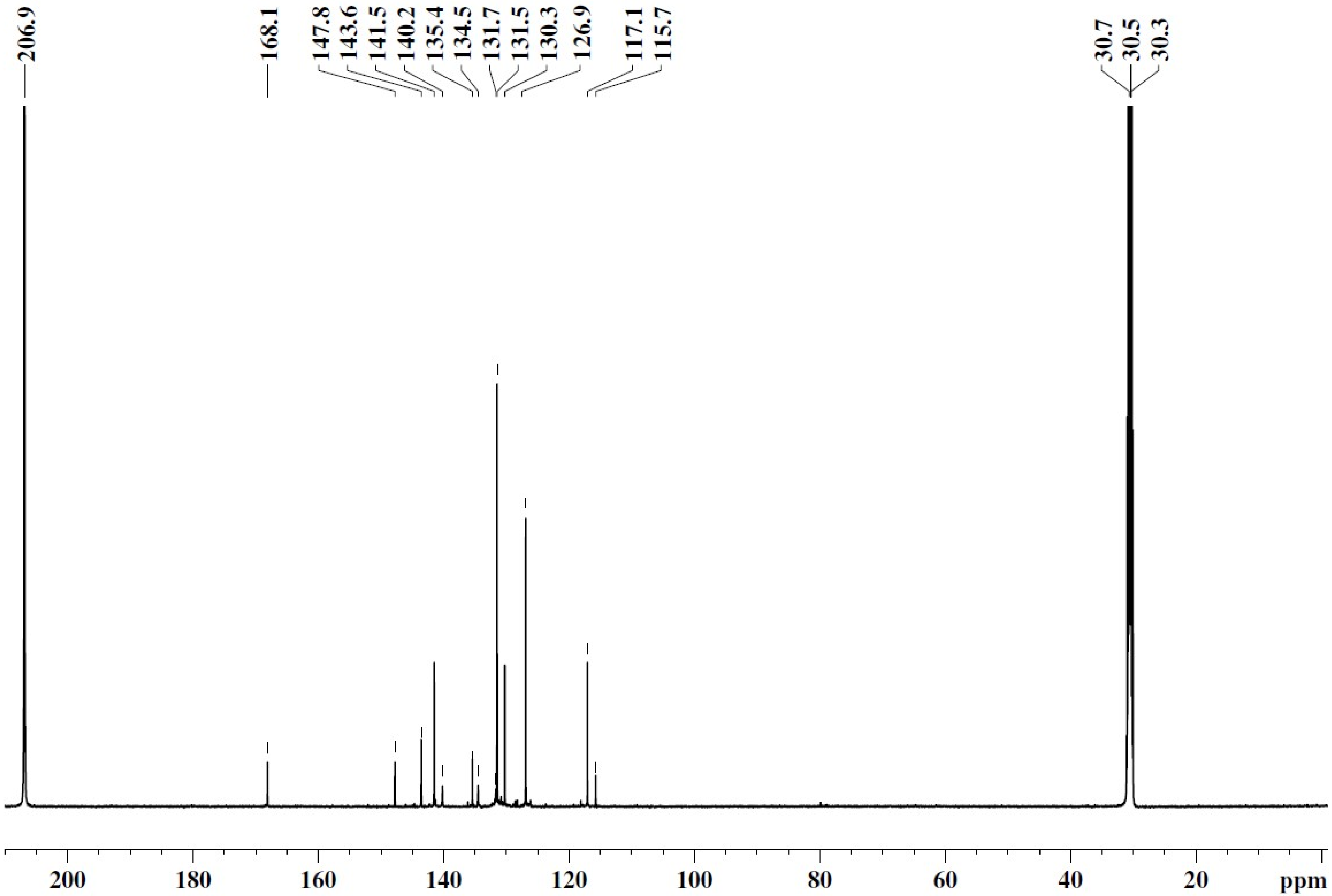

Figure S8. $^{13}C\{1H\}$ NMR (acetone-d_6, 126 MHz, 303 K) of compound **3d**.

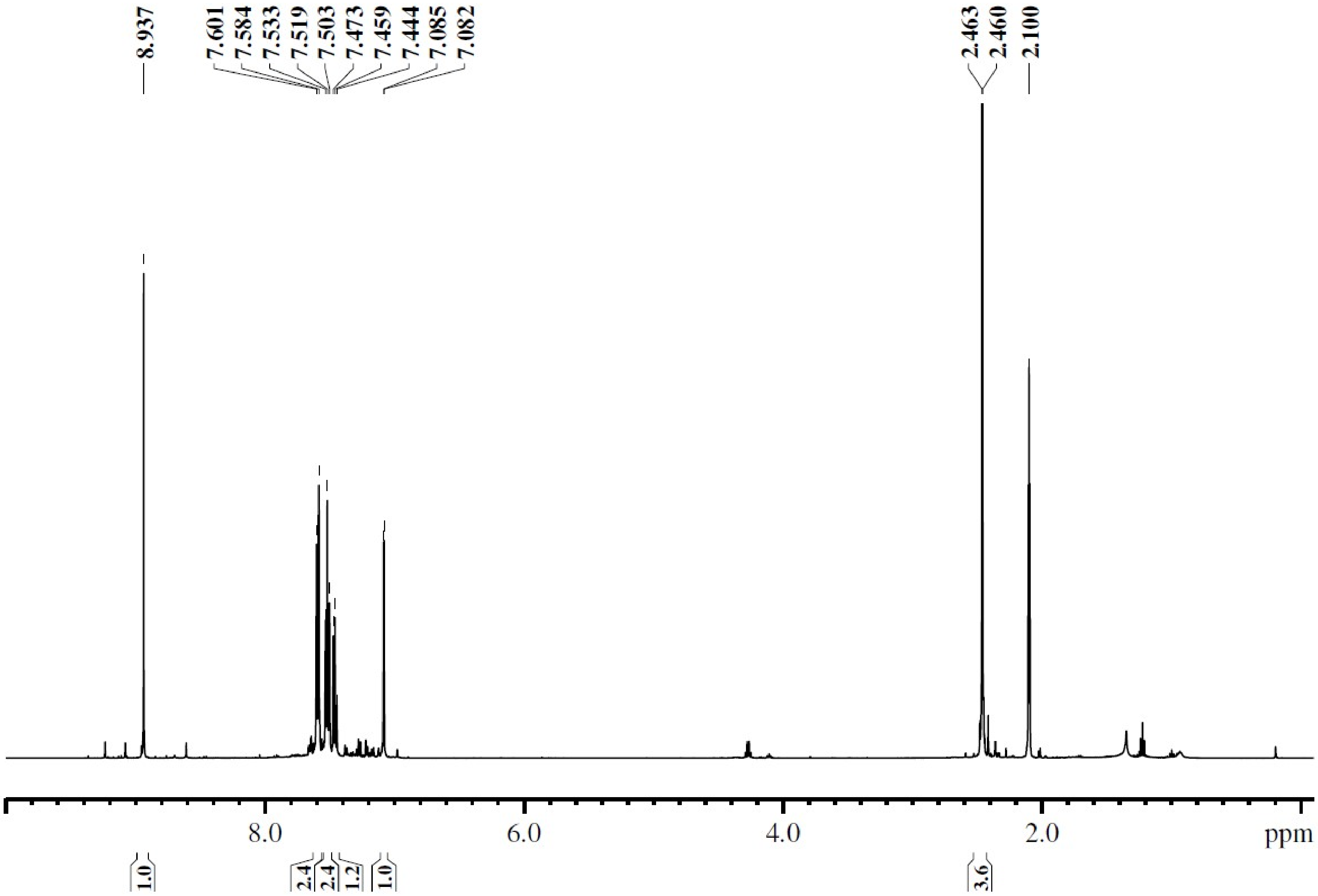

Figure S9. ^{1}H NMR (acetone-d_6, 500 MHz, 303 K) of compound **3e**.

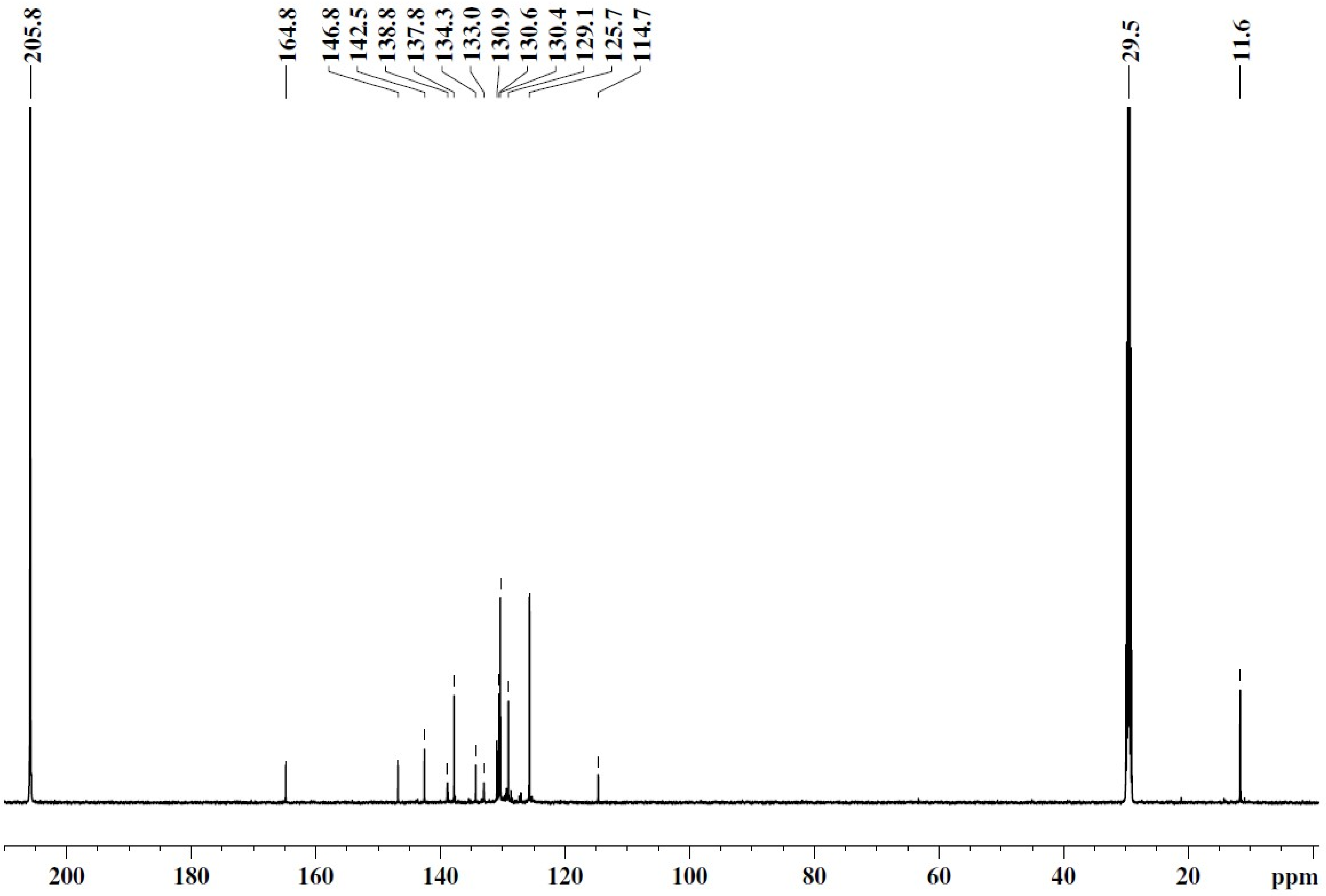

Figure S10. $^{13}C\{1H\}$ NMR (acetone-d_6, 126 MHz, 303 K) of compound **3e**.

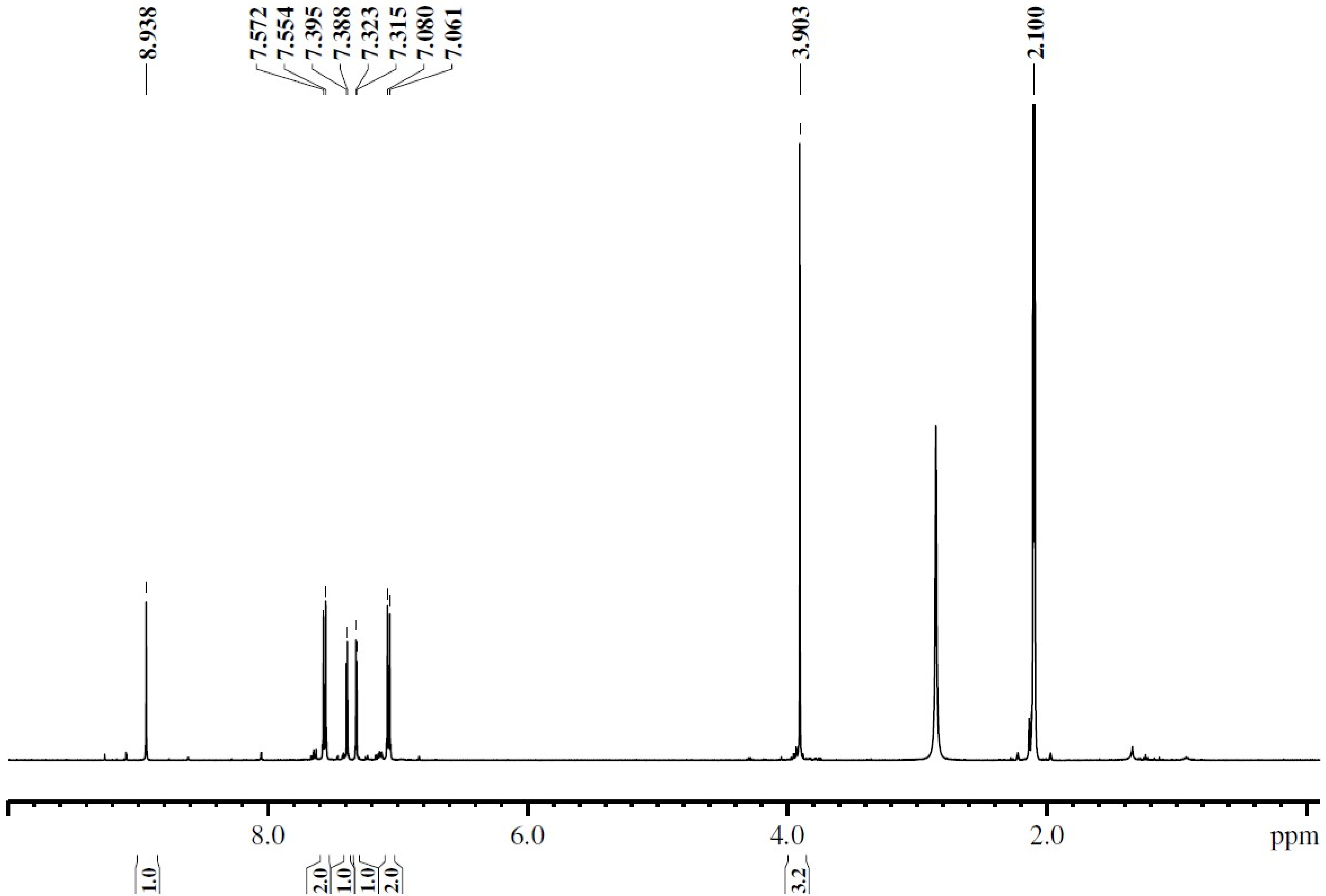

Figure S11. ^{1}H NMR (acetone-d_6, 500 MHz, 303 K) of compound **3f**.

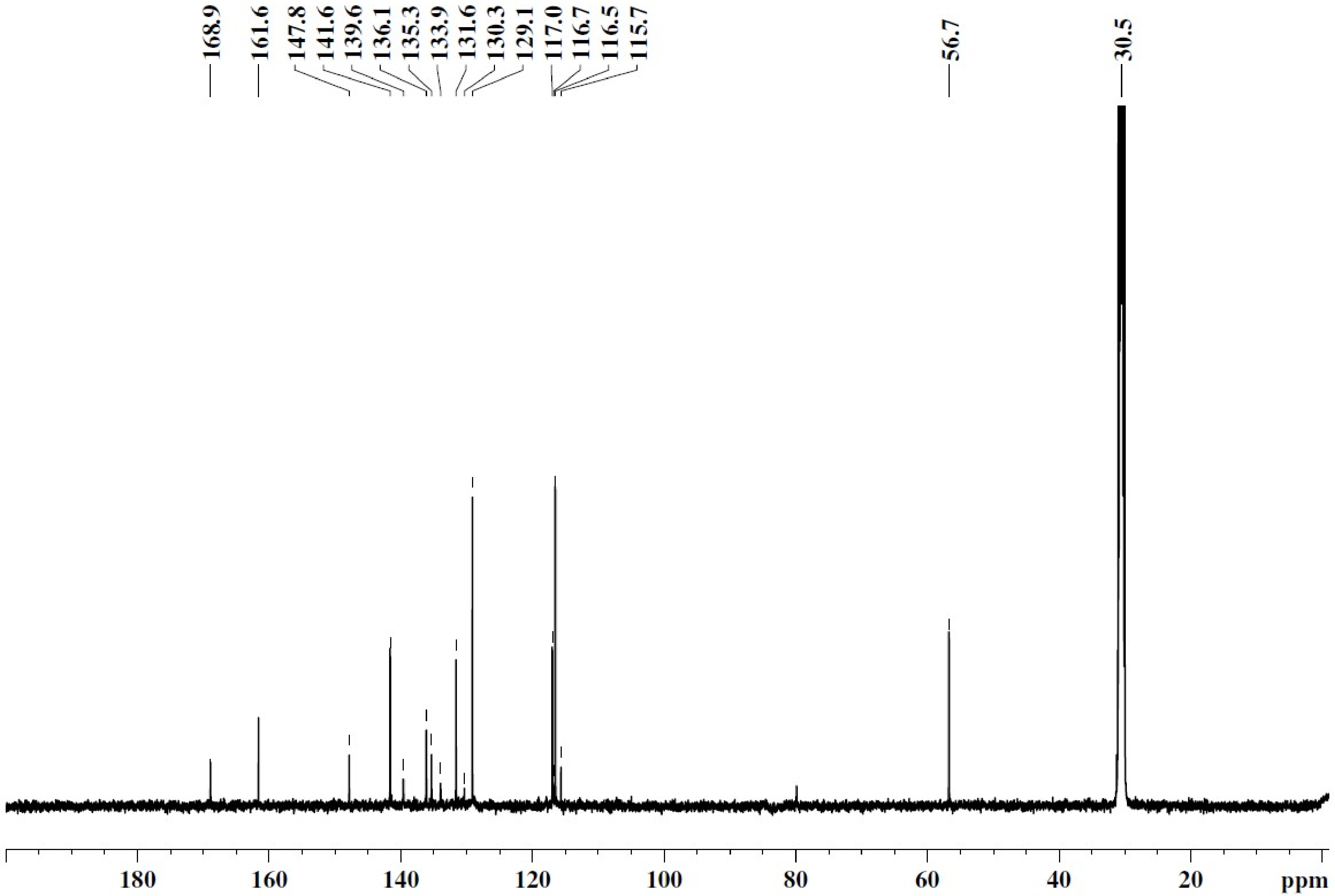

Figure S12. $^{13}C\{1H\}$ NMR (acetone-d_6, 126 MHz, 303 K) of compound **3f**.

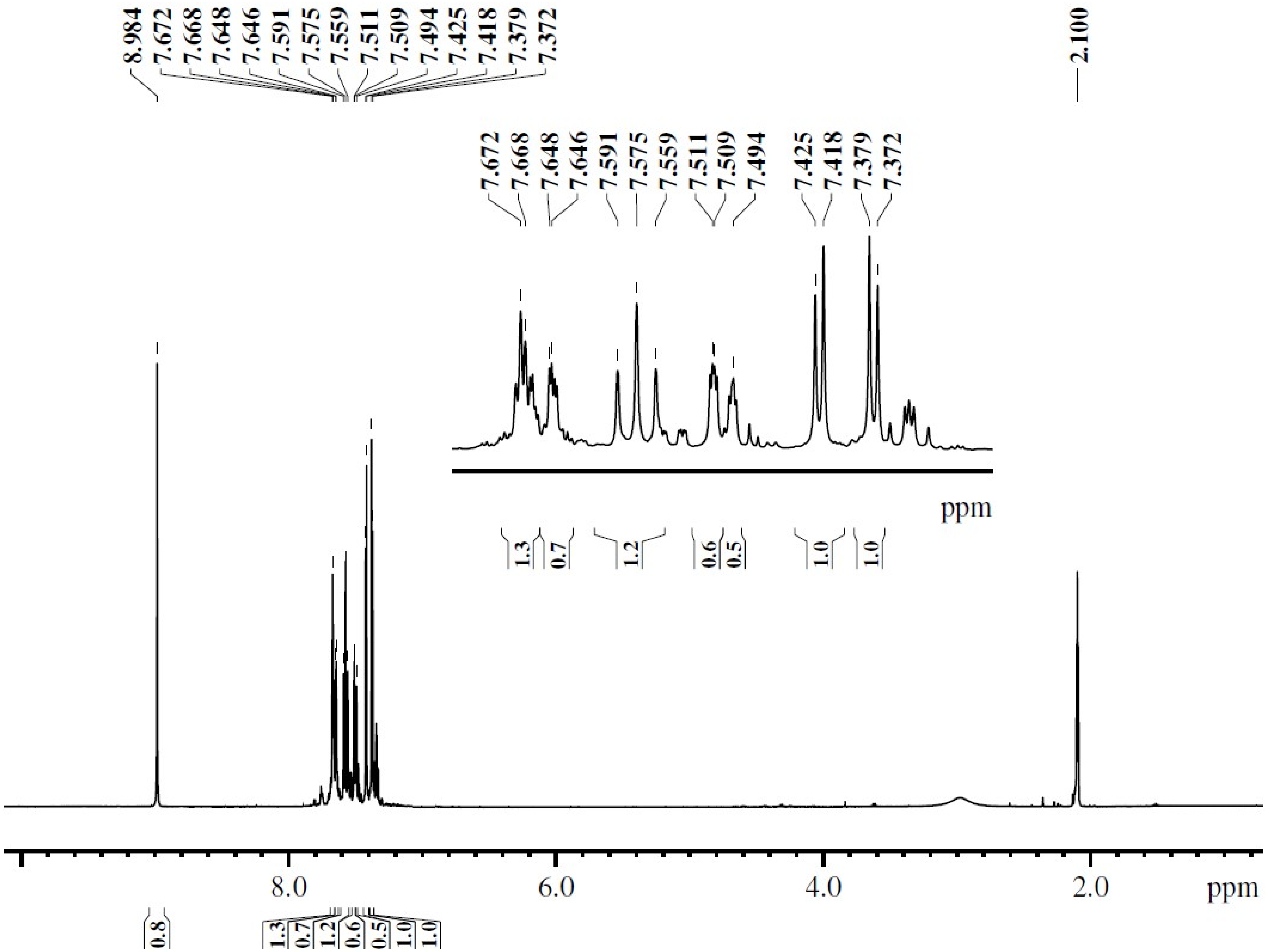

Figure S13. ^{1}H NMR (acetone-d$_6$, 500 MHz, 303 K) of compound **3g**.

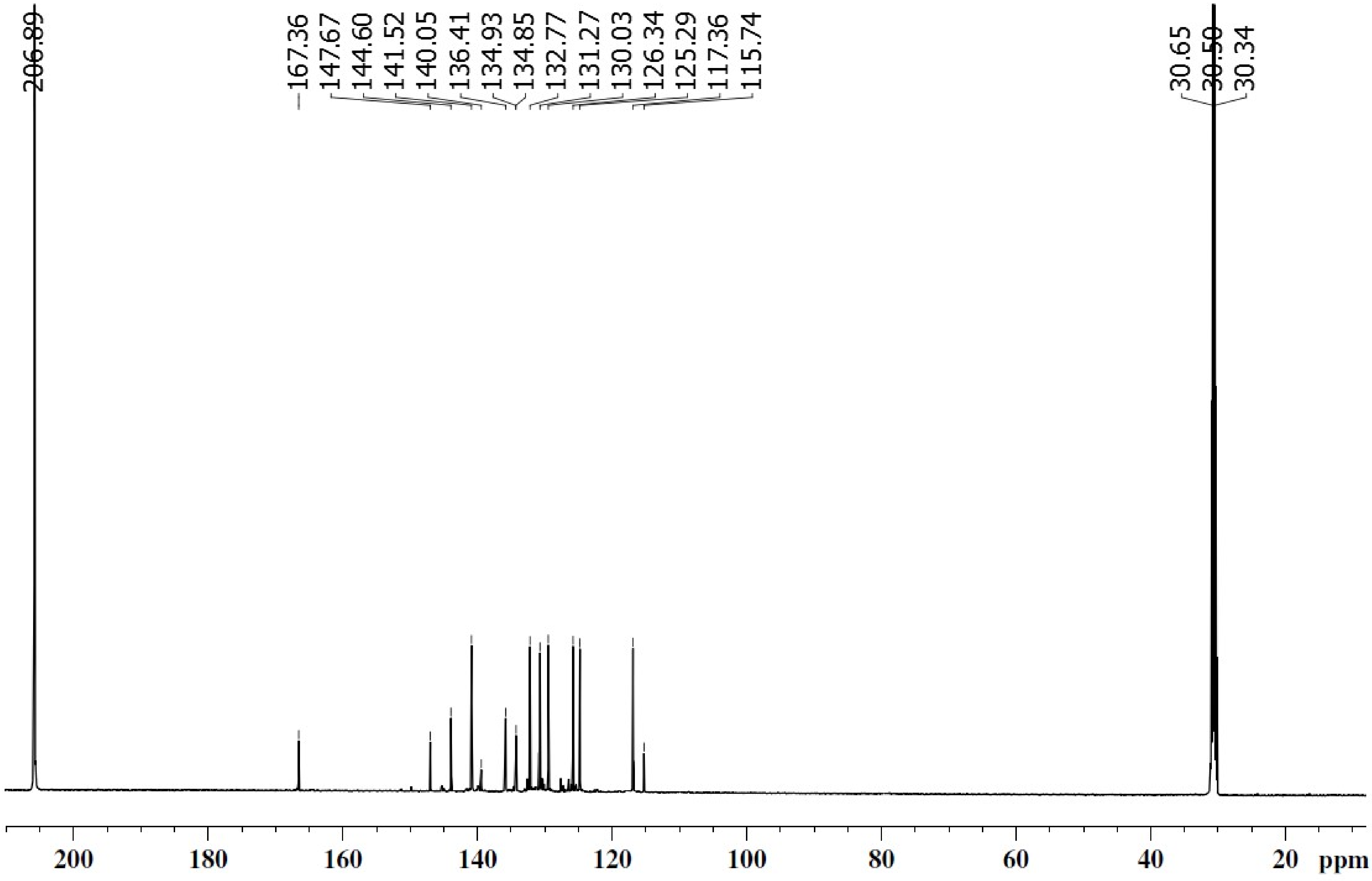

Figure S14. $^{13}C\{1H\}$ NMR (acetone-d_6, 126 MHz, 303 K) of compound **3g**.

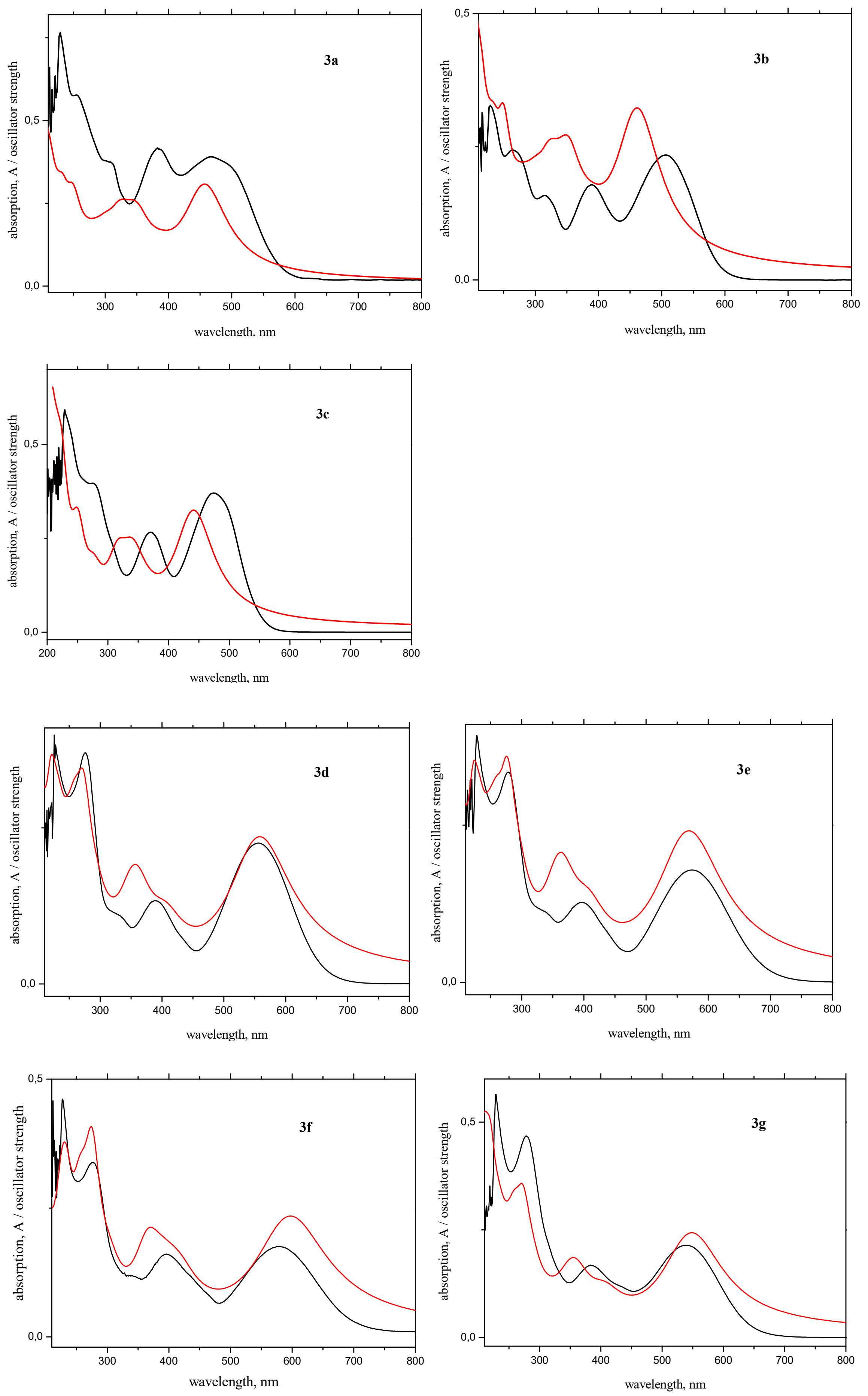

Figure S15. Experimental (black) vs theoretical (red) UV/Vis spectra of **3a** – **3g**.

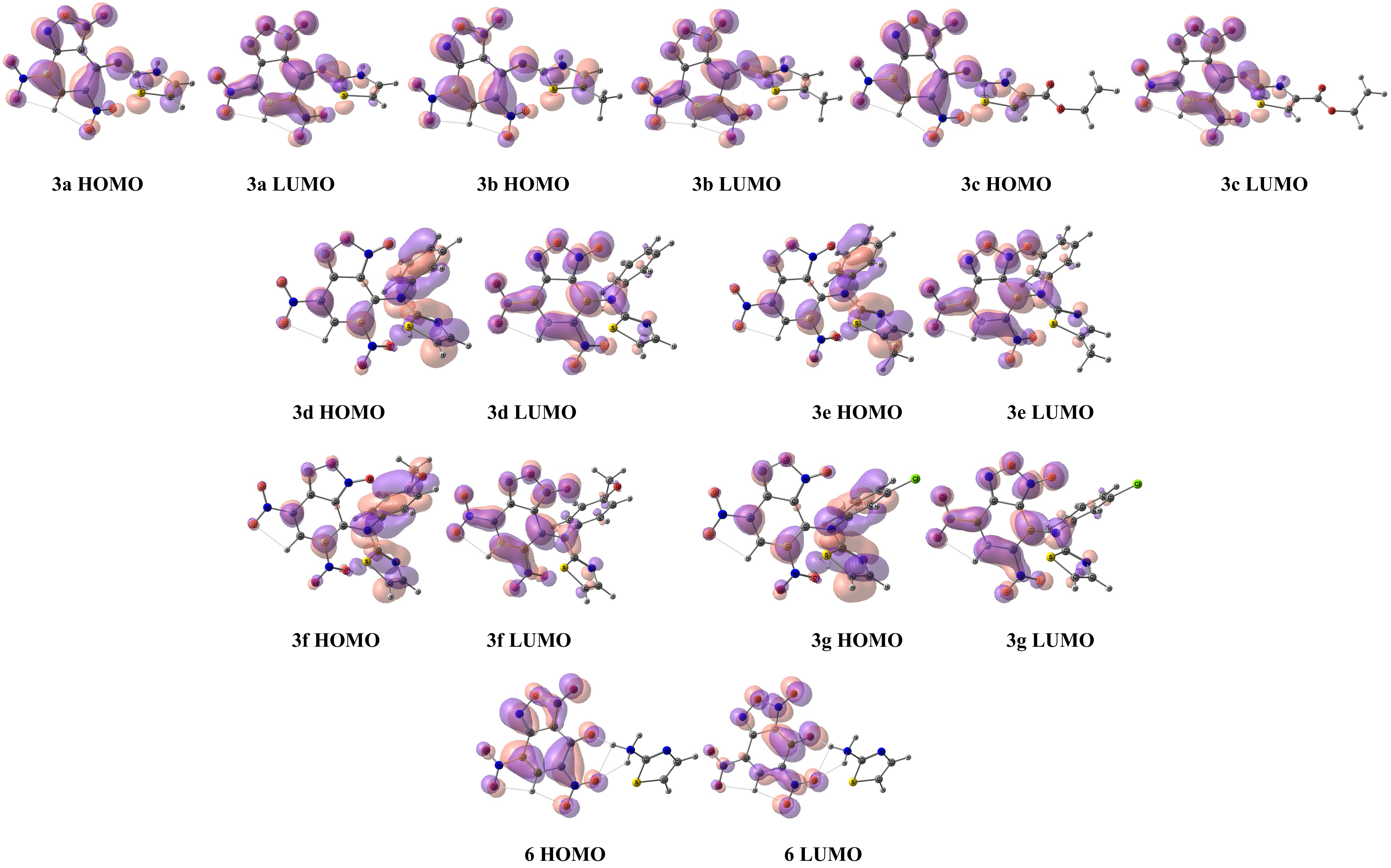

Figure S16. Frontier orbitals of **3a** – **3g** and **6**.

Molecule 2

1

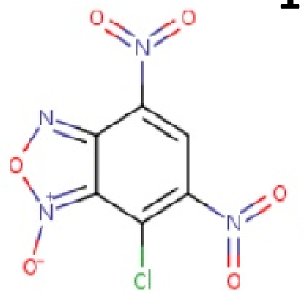

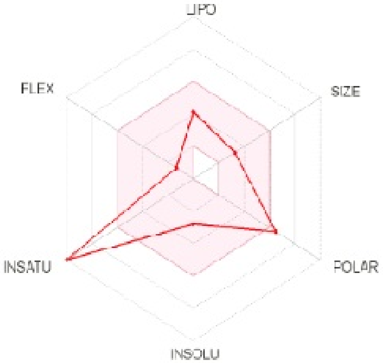

SMILES O=N(=O)c1cc(N(=O)=O)c2c(c1Cl)[n+]([O-])on2

Physicochemical Properties	
Formula	C6HClN4O6
Molecular weight	260.55 g/mol
Num. heavy atoms	17
Num. arom. heavy atoms	9
Fraction Csp3	0.00
Num. rotatable bonds	2
Num. H-bond acceptors	7
Num. H-bond donors	0
Molar Refractivity	57.82
TPSA	143.13 Å²

Lipophilicity	
Log $P_{o/w}$ (iLOGP)	-0.69
Log $P_{o/w}$ (XLOGP3)	1.70
Log $P_{o/w}$ (WLOGP)	1.98
Log $P_{o/w}$ (MLOGP)	-0.17
Log $P_{o/w}$ (SILICOS-IT)	2.60
Consensus Log $P_{o/w}$	0.04

Water Solubility	
Log *S* (ESOL)	-2.79
Solubility	4.26e-01 mg/ml ; 1.64e-03 mol/l
Class	Soluble
Log *S* (Ali)	-4.32
Solubility	1.24e-02 mg/ml ; 4.78e-05 mol/l
Class	Moderately soluble
Log *S* (SILICOS-IT)	-1.48
Solubility	8.57e+00 mg/ml ; 3.29e-02 mol/l
Class	Soluble

Pharmacokinetics	
GI absorption	Low
BBB permeant	No
P-gp substrate	No
CYP1A2 inhibitor	No
CYP2C19 inhibitor	Yes
CYP2C9 inhibitor	No
CYP2D6 inhibitor	No
CYP3A4 inhibitor	No
Log K_p (skin permeation)	-6.68 cm/s

Druglikeness	
Lipinski	Yes; 0 violation
Ghose	No; 1 violation: #atoms<20
Veber	No; 1 violation: TPSA>140
Egan	No; 1 violation: TPSA>131.6
Muegge	Yes
Bioavailability Score	0.55

Medicinal Chemistry	
PAINS	0 alert
Brenk	2 alerts: N_oxide, nitro_group
Leadlikeness	Yes
Synthetic accessibility	2.87

Figure S17. ADMET prediction for compound **1**

Molecule 3

3a

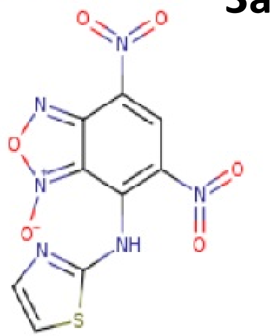

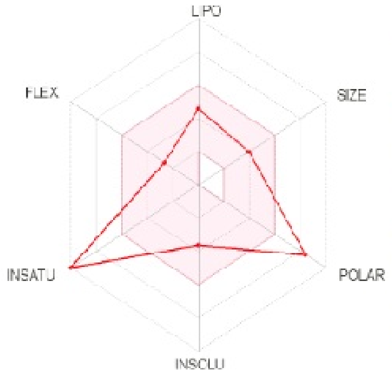

SMILES O=N(=O)c1cc(N(=O)=O)c2c(c1Nc1nccs1)[n+]([O-])on2

Physicochemical Properties	
Formula	C9H4N6O6S
Molecular weight	324.23 g/mol
Num. heavy atoms	22
Num. arom. heavy atoms	14
Fraction Csp3	0.00
Num. rotatable bonds	4
Num. H-bond acceptors	8
Num. H-bond donors	1
Molar Refractivity	78.03
TPSA	196.29 Å²

Lipophilicity	
Log $P_{o/w}$ (iLOGP)	0.64
Log $P_{o/w}$ (XLOGP3)	2.53
Log $P_{o/w}$ (WLOGP)	2.52
Log $P_{o/w}$ (MLOGP)	0.54
Log $P_{o/w}$ (SILICOS-IT)	-2.52
Consensus Log $P_{o/w}$	0.53

Water Solubility	
Log *S* (ESOL)	-3.65
Solubility	7.24e-02 mg/ml ; 2.23e-04 mol/l
Class	Soluble
Log *S* (Ali)	-6.30
Solubility	1.63e-04 mg/ml ; 5.03e-07 mol/l
Class	Poorly soluble
Log *S* (SILICOS-IT)	-2.31
Solubility	1.60e+00 mg/ml ; 4.94e-03 mol/l
Class	Soluble

Pharmacokinetics	
GI absorption	Low
BBB permeant	No
P-gp substrate	Yes
CYP1A2 inhibitor	No
CYP2C19 inhibitor	Yes
CYP2C9 inhibitor	No
CYP2D6 inhibitor	No
CYP3A4 inhibitor	No
Log K_p (skin permeation)	-6.48 cm/s

Druglikeness	
Lipinski	Yes; 1 violation: NorO>10
Ghose	Yes
Veber	No; 1 violation: TPSA>140
Egan	No; 1 violation: TPSA>131.6
Muegge	No; 1 violation: TPSA>150
Bioavailability Score	0.55

Medicinal Chemistry	
PAINS	0 alert
Brenk	2 alerts: N_oxide, nitro_group
Leadlikeness	Yes
Synthetic accessibility	3.26

Figure S18. ADMET prediction for compound **3a**

Molecule 4

3b

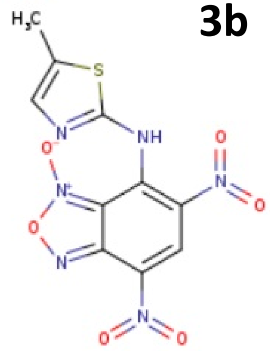

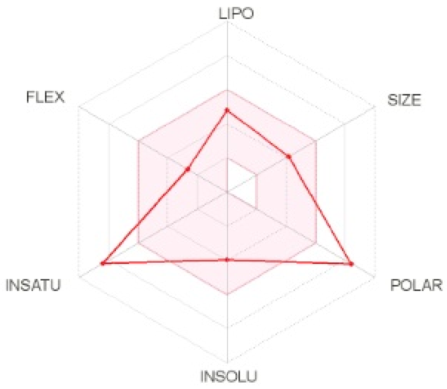

SMILES Cc1cnc(s1)Nc1c(cc(c2c1[n+]([O-])on2)N(=O)=O)N(=O)=O

Physicochemical Properties	
Formula	C10H6N6O6S
Molecular weight	338.26 g/mol
Num. heavy atoms	23
Num. arom. heavy atoms	14
Fraction Csp3	0.10
Num. rotatable bonds	4
Num. H-bond acceptors	8
Num. H-bond donors	1
Molar Refractivity	83.00
TPSA	196.29 Å²

Lipophilicity	
Log $P_{o/w}$ (iLOGP)	0.99
Log $P_{o/w}$ (XLOGP3)	2.93
Log $P_{o/w}$ (WLOGP)	2.83
Log $P_{o/w}$ (MLOGP)	-0.24
Log $P_{o/w}$ (SILICOS-IT)	-2.07
Consensus Log $P_{o/w}$	0.89

Water Solubility	
Log *S* (ESOL)	-3.97
Solubility	3.63e-02 mg/ml ; 1.07e-04 mol/l
Class	Soluble
Log *S* (Ali)	-6.71
Solubility	6.54e-05 mg/ml ; 1.93e-07 mol/l
Class	Poorly soluble
Log *S* (SILICOS-IT)	-2.69
Solubility	6.99e-01 mg/ml ; 2.07e-03 mol/l
Class	Soluble

Pharmacokinetics	
GI absorption	Low
BBB permeant	No
P-gp substrate	Yes
CYP1A2 inhibitor	No
CYP2C19 inhibitor	Yes
CYP2C9 inhibitor	No
CYP2D6 inhibitor	No
CYP3A4 inhibitor	No
Log K_p (skin permeation)	-6.28 cm/s

Druglikeness	
Lipinski	Yes; 1 violation: NorO>10
Ghose	Yes
Veber	No; 1 violation: TPSA>140
Egan	No; 1 violation: TPSA>131.6
Muegge	No; 1 violation: TPSA>150
Bioavailability Score	0.55

Medicinal Chemistry	
PAINS	0 alert
Brenk	2 alerts: N_oxide, nitro_group
Leadlikeness	Yes
Synthetic accessibility	3.33

Figure S19. ADMET prediction for compound **3b**

Molecule 5

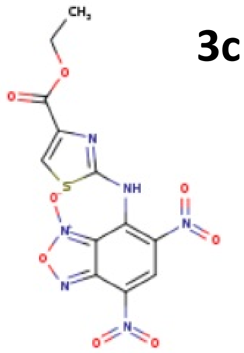

3c

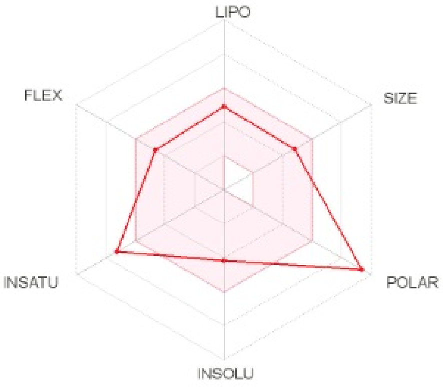

SMILES CCOC(=O)c1csc(n1)Nc1c(cc(c2c1[n+]([O-])on2)N(=O)=O)N(=O)=O

Physicochemical Properties	
Formula	C12H8N6O8S
Molecular weight	396.29 g/mol
Num. heavy atoms	27
Num. arom. heavy atoms	14
Fraction Csp3	0.17
Num. rotatable bonds	7
Num. H-bond acceptors	10
Num. H-bond donors	1
Molar Refractivity	94.12
TPSA	222.59 Å²

Lipophilicity	
Log $P_{o/w}$ (iLOGP)	1.12
Log $P_{o/w}$ (XLOGP3)	3.08
Log $P_{o/w}$ (WLOGP)	2.70
Log $P_{o/w}$ (MLOGP)	-0.15
Log $P_{o/w}$ (SILICOS-IT)	-2.25
Consensus Log $P_{o/w}$	0.90

Water Solubility	
Log *S* (ESOL)	-4.16
Solubility	2.75e-02 mg/ml ; 6.93e-05 mol/l
Class	Moderately soluble
Log *S* (Ali)	-7.42
Solubility	1.50e-05 mg/ml ; 3.79e-08 mol/l
Class	Poorly soluble
Log *S* (SILICOS-IT)	-2.74
Solubility	7.28e-01 mg/ml ; 1.84e-03 mol/l
Class	Soluble

Pharmacokinetics	
GI absorption	Low
BBB permeant	No
P-gp substrate	Yes
CYP1A2 inhibitor	No
CYP2C19 inhibitor	Yes
CYP2C9 inhibitor	No
CYP2D6 inhibitor	No
CYP3A4 inhibitor	Yes
Log K_p (skin permeation)	-6.53 cm/s

Druglikeness	
Lipinski	Yes; 1 violation: NorO>10
Ghose	Yes
Veber	No; 1 violation: TPSA>140
Egan	No; 1 violation: TPSA>131.6
Muegge	No; 1 violation: TPSA>150
Bioavailability Score	0.55

Medicinal Chemistry	
PAINS	0 alert
Brenk	2 alerts: N_oxide, nitro_group
Leadlikeness	No; 1 violation: MW>350
Synthetic accessibility	3.60

Figure S20. ADMET prediction for compound **3c**

Molecule 6

3d

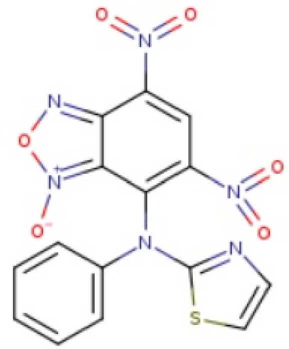

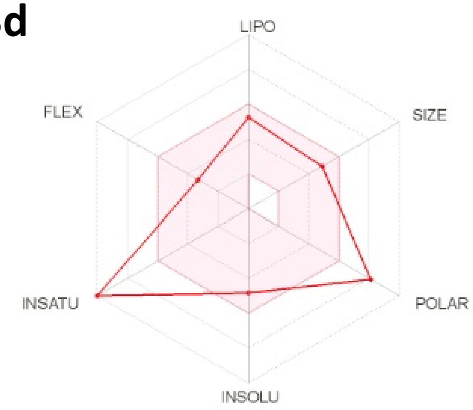

SMILES O=N(=O)c1cc(N(=O)=O)c2c(c1N(c1nccs1)c1ccccc1)[n+]([O-])on2

Physicochemical Properties	
Formula	C15H8N6O6S
Molecular weight	400.33 g/mol
Num. heavy atoms	28
Num. arom. heavy atoms	20
Fraction Csp3	0.00
Num. rotatable bonds	5
Num. H-bond acceptors	8
Num. H-bond donors	0
Molar Refractivity	103.17
TPSA	187.50 Å²

Lipophilicity	
Log $P_{o/w}$ (iLOGP)	1.26
Log $P_{o/w}$ (XLOGP3)	3.68
Log $P_{o/w}$ (WLOGP)	4.25
Log $P_{o/w}$ (MLOGP)	1.19
Log $P_{o/w}$ (SILICOS-IT)	-1.75
Consensus Log $P_{o/w}$	1.73

Water Solubility	
Log S (ESOL)	-4.84
Solubility	5.80e-03 mg/ml ; 1.45e-05 mol/l
Class	Moderately soluble
Log S (Ali)	-7.31
Solubility	1.97e-05 mg/ml ; 4.93e-08 mol/l
Class	Poorly soluble
Log S (SILICOS-IT)	-4.05
Solubility	3.55e-02 mg/ml ; 8.87e-05 mol/l
Class	Moderately soluble

Pharmacokinetics	
GI absorption	Low
BBB permeant	No
P-gp substrate	No
CYP1A2 inhibitor	No
CYP2C19 inhibitor	Yes
CYP2C9 inhibitor	Yes
CYP2D6 inhibitor	No
CYP3A4 inhibitor	No
Log K_p (skin permeation)	-6.13 cm/s

Druglikeness	
Lipinski	Yes; 1 violation: NorO>10
Ghose	Yes
Veber	No; 1 violation: TPSA>140
Egan	No; 1 violation: TPSA>131.6
Muegge	No; 1 violation: TPSA>150
Bioavailability Score	0.55

Medicinal Chemistry	
PAINS	0 alert
Brenk	2 alerts: N_oxide, nitro_group
Leadlikeness	No; 2 violations: MW>350, XLOGP3>3.5
Synthetic accessibility	3.69

Figure S21. ADMET prediction for compound **3d**

Molecule 9

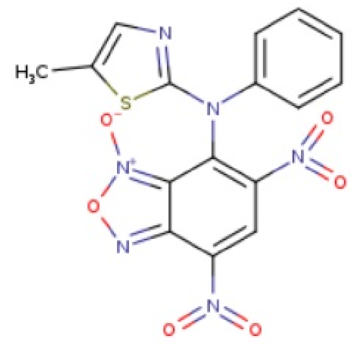

3e

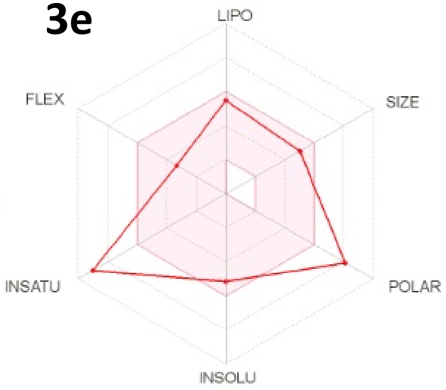

SMILES Cc1cnc(s1)N(c1c(cc(c2c1[n+]([O-])on2)N(=O)=O)N(=O)=O)c1ccccc1

Physicochemical Properties	
Formula	C16H10N6O6S
Molecular weight	414.35 g/mol
Num. heavy atoms	29
Num. arom. heavy atoms	20
Fraction Csp3	0.06
Num. rotatable bonds	5
Num. H-bond acceptors	8
Num. H-bond donors	0
Molar Refractivity	108.14
TPSA	187.50 Å²

Lipophilicity	
Log $P_{o/w}$ (iLOGP)	1.60
Log $P_{o/w}$ (XLOGP3)	4.08
Log $P_{o/w}$ (WLOGP)	4.56
Log $P_{o/w}$ (MLOGP)	1.44
Log $P_{o/w}$ (SILICOS-IT)	-1.25
Consensus Log $P_{o/w}$	2.08

Water Solubility	
Log *S* (ESOL)	-5.16
Solubility	2.87e-03 mg/ml ; 6.92e-06 mol/l
Class	Moderately soluble
Log *S* (Ali)	-7.72
Solubility	7.85e-06 mg/ml ; 1.89e-08 mol/l
Class	Poorly soluble
Log *S* (SILICOS-IT)	-4.43
Solubility	1.55e-02 mg/ml ; 3.74e-05 mol/l
Class	Moderately soluble

Pharmacokinetics	
GI absorption	Low
BBB permeant	No
P-gp substrate	No
CYP1A2 inhibitor	No
CYP2C19 inhibitor	Yes
CYP2C9 inhibitor	Yes
CYP2D6 inhibitor	No
CYP3A4 inhibitor	No
Log K_p (skin permeation)	-5.93 cm/s

Druglikeness	
Lipinski	Yes; 1 violation: NorO>10
Ghose	Yes
Veber	No; 1 violation: TPSA>140
Egan	No; 1 violation: TPSA>131.6
Muegge	No; 1 violation: TPSA>150
Bioavailability Score	0.55

Medicinal Chemistry	
PAINS	0 alert
Brenk	2 alerts: N_oxide, nitro_group
Leadlikeness	No; 2 violations: MW>350, XLOGP3>3.5
Synthetic accessibility	3.76

Figure S22. ADMET prediction for compound **3e**

swissadme.ch/index.php

Molecule 1

3f

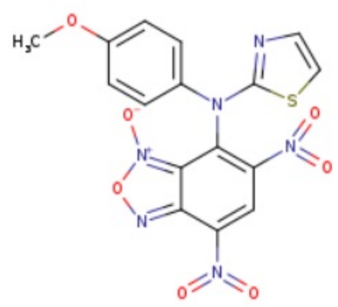

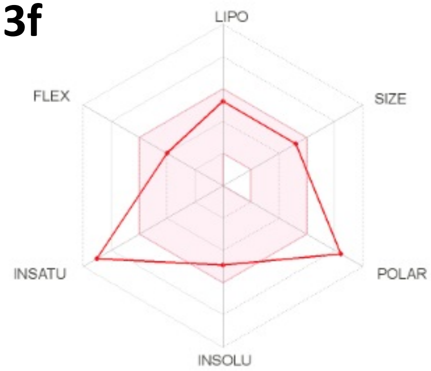

SMILES COc1ccc(cc1)N(c1c(cc(c2c1[n+]([O-])on2)N(=O)=O)N(=O)=O)c1nccs1

Physicochemical Properties	
Formula	C16H10N6O7S
Molecular weight	430.35 g/mol
Num. heavy atoms	30
Num. arom. heavy atoms	20
Fraction Csp3	0.06
Num. rotatable bonds	6
Num. H-bond acceptors	9
Num. H-bond donors	0
Molar Refractivity	109.66
TPSA	196.73 Å²

Lipophilicity	
Log $P_{o/w}$ (iLOGP)	1.51
Log $P_{o/w}$ (XLOGP3)	3.65
Log $P_{o/w}$ (WLOGP)	4.26
Log $P_{o/w}$ (MLOGP)	0.95
Log $P_{o/w}$ (SILICOS-IT)	-1.70
Consensus Log $P_{o/w}$	1.74

Water Solubility	
Log *S* (ESOL)	-4.91
Solubility	5.36e-03 mg/ml ; 1.24e-05 mol/l
Class	Moderately soluble
Log *S* (Ali)	-7.47
Solubility	1.46e-05 mg/ml ; 3.39e-08 mol/l
Class	Poorly soluble
Log *S* (SILICOS-IT)	-4.15
Solubility	3.03e-02 mg/ml ; 7.03e-05 mol/l
Class	Moderately soluble

Pharmacokinetics	
GI absorption	Low
BBB permeant	No
P-gp substrate	No
CYP1A2 inhibitor	No
CYP2C19 inhibitor	Yes
CYP2C9 inhibitor	Yes
CYP2D6 inhibitor	No
CYP3A4 inhibitor	Yes
Log K_p (skin permeation)	-6.33 cm/s

Druglikeness	
Lipinski	Yes; 1 violation: NorO>10
Ghose	Yes
Veber	No; 1 violation: TPSA>140
Egan	No; 1 violation: TPSA>131.6
Muegge	No; 1 violation: TPSA>150
Bioavailability Score	0.55

Medicinal Chemistry	
PAINS	0 alert
Brenk	2 alerts: N_oxide, nitro_group
Leadlikeness	No; 2 violations: MW>350, XLOGP3>3.5
Synthetic accessibility	3.68

Figure S23. ADMET prediction for compound **3f**

Molecule 8

3g

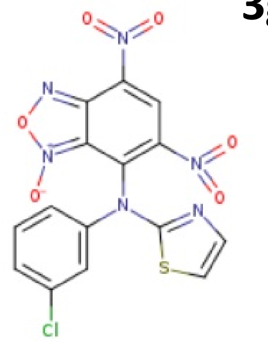

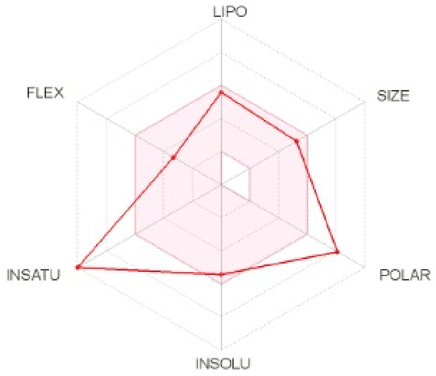

SMILES: Clc1cccc(c1)N(c1c(cc(c2c1[n+]([O-])on2)N(=O)=O)N(=O)=O)c1nccs1

Physicochemical Properties	
Formula	C15H7ClN6O6S
Molecular weight	434.77 g/mol
Num. heavy atoms	29
Num. arom. heavy atoms	20
Fraction Csp3	0.00
Num. rotatable bonds	5
Num. H-bond acceptors	8
Num. H-bond donors	0
Molar Refractivity	108.18
TPSA	187.50 Å²

Lipophilicity	
Log $P_{o/w}$ (iLOGP)	0.76
Log $P_{o/w}$ (XLOGP3)	4.30
Log $P_{o/w}$ (WLOGP)	4.90
Log $P_{o/w}$ (MLOGP)	1.70
Log $P_{o/w}$ (SILICOS-IT)	-1.10
Consensus Log $P_{o/w}$	2.11

Water Solubility	
Log *S* (ESOL)	-5.42
Solubility	1.63e-03 mg/ml ; 3.76e-06 mol/l
Class	Moderately soluble
Log *S* (Ali)	-7.95
Solubility	4.87e-06 mg/ml ; 1.12e-08 mol/l
Class	Poorly soluble
Log *S* (SILICOS-IT)	-4.64
Solubility	1.00e-02 mg/ml ; 2.31e-05 mol/l
Class	Moderately soluble

Pharmacokinetics	
GI absorption	Low
BBB permeant	No
P-gp substrate	No
CYP1A2 inhibitor	No
CYP2C19 inhibitor	Yes
CYP2C9 inhibitor	No
CYP2D6 inhibitor	No
CYP3A4 inhibitor	No
Log K_p (skin permeation)	-5.90 cm/s

Druglikeness	
Lipinski	Yes; 1 violation: NorO>10
Ghose	Yes
Veber	No; 1 violation: TPSA>140
Egan	No; 1 violation: TPSA>131.6
Muegge	No; 1 violation: TPSA>150
Bioavailability Score	0.55

Medicinal Chemistry	
PAINS	0 alert
Brenk	2 alerts: N_oxide, nitro_group
Leadlikeness	No; 2 violations: MW>350, XLOGP3>3.5
Synthetic accessibility	3.64

Figure S24. ADMET prediction for compound **3g**
